# Mining protein loops using a structural alphabet and statistical exceptionality

**DOI:** 10.1186/1471-2105-11-75

**Published:** 2010-02-04

**Authors:** Leslie Regad, Juliette Martin, Gregory Nuel, Anne-Claude Camproux

**Affiliations:** 1 MTi, Inserm UMR-S 973, Université Paris Diderot- Paris 7, Paris, F-75205 Cedex 13, France; 2 Unite Mathématiques Informatique et Génome UR1077, INRA, Jouy-en-Josas, F-78350, France; 3 Université Lyon 1, IFR 128, CNRS, UMR 5086, IBCP, Institut de Biologie et Chimie des Protéines, Lyon, F-69367, France; 4 MAP5, UMR CNRS 8145, Université Paris-Descartes,Paris, F-75006, France

## Abstract

**Background:**

Protein loops encompass 50% of protein residues in available three-dimensional structures. These regions are often involved in protein functions, e.g. binding site, catalytic pocket... However, the description of protein loops with conventional tools is an uneasy task. Regular secondary structures, helices and strands, have been widely studied whereas loops, because they are highly variable in terms of sequence and structure, are difficult to analyze. Due to data sparsity, long loops have rarely been systematically studied.

**Results:**

We developed a simple and accurate method that allows the description and analysis of the structures of short and long loops using structural motifs without restriction on loop length. This method is based on the structural alphabet HMM-SA. HMM-SA allows the simplification of a three-dimensional protein structure into a one-dimensional string of states, where each state is a four-residue prototype fragment, called structural letter. The difficult task of the structural grouping of huge data sets is thus easily accomplished by handling structural letter strings as in conventional protein sequence analysis. We systematically extracted all seven-residue fragments in a bank of 93000 protein loops and grouped them according to the structural-letter sequence, named structural word. This approach permits a systematic analysis of loops of all sizes since we consider the structural motifs of seven residues rather than complete loops. We focused the analysis on highly recurrent words of loops (observed more than 30 times). Our study reveals that 73% of loop-lengths are covered by only 3310 highly recurrent structural words out of 28274 observed words). These structural words have low structural variability (mean RMSd of 0.85 Å). As expected, half of these motifs display a flanking-region preference but interestingly, two thirds are shared by short (less than 12 residues) and long loops. Moreover, half of recurrent motifs exhibit a significant level of amino-acid conservation with at least four significant positions and 87% of long loops contain at least one such word. We complement our analysis with the detection of statistically over-represented patterns of structural letters as in conventional DNA sequence analysis. About 30% (930) of structural words are over-represented, and cover about 40% of loop lengths. Interestingly, these words exhibit lower structural variability and higher sequential specificity, suggesting structural or functional constraints.

**Conclusions:**

We developed a method to systematically decompose and study protein loops using recurrent structural motifs. This method is based on the structural alphabet HMM-SA and not on structural alignment and geometrical parameters. We extracted meaningful structural motifs that are found in both short and long loops. To our knowledge, it is the first time that pattern mining helps to increase the signal-to-noise ratio in protein loops. This finding helps to better describe protein loops and might permit to decrease the complexity of long-loop analysis. Detailed results are available at http://www.mti.univ-paris-diderot.fr/publication/supplementary/2009/ACCLoop/.

## Background

Protein structures are classically described using secondary structures: *α*-helices, *β*-strands and loops, also called coils. This third class is a default description, which denotes all residues that are not involved in periodic local structures, helices or strands. On average, protein loops encompass 50% of residues. Protein loops are often involved in protein functions [1]. They participate in active sites of enzymes [2] and in molecular recognition [3,4]. They are often the place of binding sites: for example, the ATP and GTP-binding site (P-loop motif) and the calcium-binding site (EF-hand motif) are found in loops [5-8]. The description and analysis of protein loops have been the subject of many studies. Protein loops were first seen as random because they are highly variable in terms of sequence and structure and are subject to frequent insertions and deletions [9,10]. Because of their large variability, loops are the protein regions which are the most difficult to analyze and modelize. Indeed, in protein models, loops, and more particularly long loops, are the place of a lot of errors.

Systematic studies actually showed that loops, even long ones, are far from random. In their study, Panchenko et al. (2004) analyzed the evolution of protein loops and identified a linear correlation between sequence similarity and average loop structural similarity in protein families [11]. They suggested that the evolution of loops is made *via *an insertion/deletion process and concluded that even longer loop regions cannot be defined as "irregular conformations" or "random coils".

The resolution of an increasing number of protein structures allowed the classification of short loops (3 to 12 residues) according to their geometry, and gave birth to several loop classification systems: Sloop [12-14], Wloop[15,16], ArchDB[17-19], Li et al. classification [20,21]. These different classification initiatives were based on different criteria such as loop length [12,14,15,17,18], flanking region type [12,14,17,18,20,21], flanking-region geometry [12,14,17,18], or loop conformation [17,18]. The majority of the resulting loop clusters presented a significant sequence signature. These classifications thus revealed the existence of recurrent loop conformations with amino-acid dependence. However, these classifications focus on short and medium loops (less than 12 residues) and do not take long loops into consideration.

Another type of studies focused on specific structural motifs extracted from loops such as *β*-turn [22-25], *β*-hairpin [26-29], helix-turn-helix [30], helix-turn-strand [31], or *ω*-loop [1,32]. The most frequent motif is *β*-turn. It corresponds to 25% of residues [33]. Other turn types have been identified such as *γ*-turn [34-36] or *α*-turns [37,38]. Recently, Golovin et al. (2008) proposed a web application that allows identifying known small structural motifs characterized by hydrogen-bonds (alpha-beta motif, asx-motif, beta-bulge, beta-bulge-loop, beta-turn, catmat, gamma-turn, nest, schellmann-loop, st-motif, st-staple, st-turn) from a query protein [8]. A database of these structural motifs extracted from a set of 400 representative proteins is now available [39]. All these studies were dedicated to particular -and known- small structural motifs, but did not perform a systematic analysis of all loops.

In a previous study, we have shown that the structural alphabet HMM-SA (Hidden Markov Model-Structural Alphabet) is an effective tool to simplify loop structures with good accuracy [40]. Structural alphabets constitute a privileged tool to discretize 3D structures including loop regions, with an accuracy that depends on the size of the fragment library [41]. HMM-SA is a collection of 27 structural prototypes of four residues called structural letters, permitting the simplification of all three-dimensional (3D) protein structures into uni-dimensional (1D) sequences of structural letters [42].

Here, we present an extensive analysis and description of both short and long loops based on the analysis of structural motifs extracted from loops. The systematic extraction of seven-residue structural motifs is based on the loop decomposition in structural letters provided by HMM-SA. Thanks to this decomposition, structural motifs are described as patterns of structural letters, called structural words. This representation as structural words permits to partition the full space of loop conformations, independently of their length, in clusters represented by distinct words. We first present general results concerning structural words: repartition of clusters and intrinsic characteristics of structural words such as structural variability and sequential specificity. Then, we present the analysis of the link between structural words and loop types. In order to gain further insight into the high complexity of loop structures, we complement our analysis with an original approach based on statistical exceptionality implemented in the SPatt software [43]. The idea is to compute, for each structural motif, a score that is a measure of its "unusualness" with respect to some background model. The goal is to assess whether some structural motifs are more or less frequent than expected. This is directly inspired by analogous studies of sequence patterns in genomes [44,45], that permitted the discovery of functional patterns such as restriction sites [46], cross-over hot spot instigator sites [47] and polyadenylation signals [48]. Finally, this systematic structural-alphabet decomposition and word analysis provide an accurate description of loops and allows extracting meaningful motifs in both short and long loops, which is an important contribution to the difficult task of long loop analysis.

## Results

We extracted all structural motifs within loops from a non-redundant data set of 8186 protein chains, using the structural alphabet HMM-SA. This alphabet is a collection of 27 prototypes of four residues, denoted [A-Z, a], based on a hidden Markov model [40,42]. It permits the encoding of a protein structure of *n *residues into a sequence of (*n *- 3) structural letters.

Loop structures extracted from our protein data set were encoded into structural-letter sequence using HMM-SA. Each encoded loop was then decomposed into overlapping structural words, i.e. series of *k *consecutive structural letters, corresponding to *k *- 3 residue fragments. Thus, structural words can be seen as a way of clustering the fragments. Each cluster of fragments is defined by a structural word. The first step of this work is the determination of the optimal length of fragments/words.

### Choice of the structural word length

The choice of the optimal length was guided by the following dilemma. On the one hand, it is desirable to consider long fragments, in order to better describe 3D conformation and capture the longest-range interactions. On the other hand, the amount of available data rapidly becomes insufficient when dealing with long fragments. To choose this optimal length, we computed the frequency of all structural words in our data set, with length from five residues (two-structural letters) to ten residues (seven-structural letters), see Additional file 1. We identified seven residues as the maximum length to avoid the problem of data sparsity. The number of different structural words sharply increases beyond that limit and 80% of structural words of 8 residues are seen at most 6 times in our data set, versus 34 times for words of 7 residues. For these reasons, we selected seven residues, i.e., four structural letters as the most meaningful length for systematic extraction.

### First Part: Global results on structural words

We systematically extracted structural words of four structural-letters from protein loops and analyzed their properties: structural variability, amino-acid specificity and preference for particular loop types.

#### Extraction of structural words from loops

The data set contained 93396 loops of minimal length seven residues (i.e. four structural letters). From these loops, we extracted 415071 overlapping seven-residue fragments. The 415071 fragments were partitioned into 28274 different four-structural-letter words, with an average cluster size of 14.7 and a high variability: standard deviation was equal to 36. As HMM-SA offers a very detailed description of loop structures, some slightly different conformations ended up in distinct clusters; our classification then disclosed with a high number (5626) of singletons, i.e. clusters containing only one fragment. However, even if we had considered X-ray structures with good resolution (better than 2.5 Å), such rare conformations might have been an artifact due to the structural flexibility of some protein regions. Indeed, protein loops are generally more flexible than regular secondary structures [49]. We tested this hypothesis using B-factors, as atoms with high B-factors are those with the largest positional uncertainty. We computed the average C*α *B-factor for all fragments in each structural word. We used the rule-of-thumb suggested in [50] and set a B-factor cut-off at 40. We found that a large proportion (28%) of singletons have an average B-factor greater than 40, compared to only 1% for structural words from clusters with more than 30 fragments. Singletons and rare conformations are thus linked to structural flexibility. In the rest of the paper, we consider a restricted set containing words seen more than 30 times (i.e., minimal cluster size set to 30), denoted *W*set_≥30_. The reason for this choice is that our goal is to perform a statistical analysis of word properties, namely structural variability and sequence specificity. Since these properties are assessed by RMSd and Z-scores extracted from sequence profiles, a sufficient number of fragments per cluster is needed. We estimated that 30 fragments were sufficient to compute mean RMSd and sequence profiles. Statistics of *W*set_≥30 _are given in Table 1. As can be seen in Table 1, *W*set_≥30 _encompass 3310 different structural words (12% of all words), and 60% of fragments.

**Table 1:** Quantification of the structural word extraction from the non-redundant data set.

Words				
Number of words	3310	166	2214	930
(%)	(11.7%)	(5.0%)	(66.9%)	(28.1%)
Number of fragments	249953	11435	129781	108737
(%)	(60.2%)	(4.6%)	(51.9%)	(43.5%)
Nb fragments/word	75.5	68.9	58.6	116.9*
All-loop coverage rate	72.7%	5.1%	46.5%	40.2%
Short-loop coverage rate	70.3%	4.4%	38.9%	39.3%
Long-loop coverage rate	74.9%	5.7%	53.9%	41.1%
Loops containing at least one word	84.8%	9.8%	60.3%	58.2%
Short loops containing at least one word	79.7%	6.1%	48.1%	49.4%
Long loops containing at least one word	97.8%	19.1%	90.9%	80.4%

1: words seen more than 30 times. 2: under-represented words, 3: non-significant words, 4: over-represented words, '*': significantly higher occurrence according to a Kruskal-Wallis test. Coverage rates are given on a per structural letter basis. Numbers within brackets denote the percentage of words/fragments with respect to the 28274 words/415071 fragments of the whole data set (column 1) and with respect to the 3310 words/249953 fragments in *W*set_≥30 _(columns 2 to 4).

#### Loop coverage by *W*set_≥30 _words

In this part, we check if the elimination of rare words does not result in (i) a dramatic diminution of loop coverage or (ii) a loss of diversity in structural families.

At first, we can observe that the selection of *W*set_≥30 _words does not favor any loop length: the distribution of loop lengths in *W*set_≥30 _is similar to the global loop-length distribution (cf. Additional file 1).

##### Global loop coverage

(cf. Materials and Methods). Words from *W*set_≥30 _encompass 60% of the fragments. However, since we extracted overlapping fragments, the coverage rate of loop structures is more than 60%: if a loop of 8 structural letters is described by two *W*set_≥30 _words on positions 1 to 4 and 5 to 8, the actual coverage is 100% even if only 2 out the 5 overlapping fragments are represented by frequent words.

Coverage rates are reported in Table 1. The limited number of words seen more than 30 times (3310) covers most loop, namely 73% of loop lengths. If we make the distinction between short loops (up to 12 residues) and long loops (longer than 12 residues), we can see that *W*set_≥30 _words cover both short and long loops. If we now consider loops that contain at least one *W*set_≥30 _word, *W*set_≥30 _words partially describe 85% of all loops -80% of short loops and 98% of long loops.

The consideration of the restricted set *W*set_≥30 _thus allowed us to get rid of clusters with high positional uncertainty while still covering a large fraction of protein loops.

##### SCOP superfamily coverage by *W*set_≥30 _words

There might be a risk that the selection of recurrent words could give preferences to loops from highly populated structural families. In order to address this problem, we assessed the coverage of *W*set_≥30 _with respect to the SCOP classification. We surveyed the SCOP classification of 8140 protein chains covered by *W*set_≥30_. The results are presented in Table 2. We identified 1493 different superfamilies in the full data set. The removal of rare words led to the elimination of 46 protein chains, and 11 SCOP superfamilies. We then checked the number of structure members in the 1485 remaining superfamilies. After the removal of words seen less than 30 times, this number was lowered for 46 superfamilies. The majority of affected superfamilies (44 among 46) lost only one member, as shown in Additional file 1. These elements suggest that the elimination of words seen less than 30 times still permits to keep a good representation of SCOP superfamilies, since 97% of initial superfamilies were unaffected. Therefore, loops from highly populated structural families are not given preferences due to the selection of recurrent words.

**Table 2:** Population of SCOP superfamilies before and after elimination of rare conformations

	All words	***W*set**_≥30_
Nb structures	8186	8140
Nb superfamilies	1493	1485
Nb superfamilies with less than 30 members	1437	1435

Consequently, we can conclude that the systematic extraction of structural words shows that most loops can be described by a limited number of frequent four-structural-letter words.

#### Structural and amino-acid conservation of words

The next step consists in analyzing the intrinsic structural and sequential properties of structural *W*set_≥30 _words. We considered the following properties: structural variability of the fragments, and dependence to their amino-acid sequence.

##### Structural properties of words

The intra-word structural variability of clusters is assessed using the average Root Mean Square deviation (RMSd_*w*_) between fragments within the same cluster. The global mean RMSd_*w *_is equal to 0.85 Å (cf. Table 3). Words exhibiting the largest structural variability include structural letters J or F. It was expected because these two letters are the most structurally variable ones [42]. We can observe that the word structural variability could be quantify by the structural-letter type. This allows avoiding the computation of RMSd and the superimposition of word fragments. This analysis shows that most words exhibit a weak structural variability.

**Table 3:** Structural and sequential properties of words in *W*set_≥30 _according to the statistical word type

Words characteristic	***W*set**_**≥30**_	***UR***_*w*_	***NS***_*w*_	***OR***_*w*_
Average RMSd_*w *_(Å)	0.85	0.94	0.89	0.74*
(± standard deviation)	(± 0.4)	(± 0.4)	(± 0.4)	(± 0.3)
Average RMSd_*dev *_(Å)	2.72	2.67	2.69	2.76
(± standard deviation)	(± 0.6)	(± 0.6)	(± 0.6)	(± 0.7)
Average*Z*_max_	10.3	9.5	8.8	14.0*
(± standard deviation)	(± 6.1)	(± 3.8)	(± 4.0)	(± 8.4)
Average nb_pos*_	3.3	3.0	2.9	4.1*
(± standard deviation)	(± 1.8)	(± 1.7)	(± 1.6)	(± 1.8)
Average *d*^*Z*-score^	31.1	29.0	27.4	39.5*
(± standard deviation)	(± 9.7)	(± 5.7)	(± 5.3)	(± 13.8)

The upper part of the table corresponds to the analysis of word structural properties. The intra-word structural variability is analysed using the Root Mean Square deviation (RMSd) between fragments corresponding to the same word (RMSd_*w*_). The inter-word structural variability is analysed using the RMSd between fragments of two different words (RMSd_*dev*_). The lower part of the table corresponds to the analysis of sequential properties of words. The intra-word amino-acid preferences of a word are analysed using *Z*_max _criterion (cf. Method section) and the number of significant position of a word (nb_pos*_). The coverage of sequential space is analysed using the Euclidian distance between *Z*-score vectors (cf. Method section) (*d*^*Z*-score^). Numbers within brackets indicate standard deviations. *: significant differences according to the Kruskal-Wallis test. The RMSd_*dev *_are computed on a subset of 890 words of *W*set_≥30_. ^*a*^: words shared by long and short loops.

##### Amino-acid preferences of words

Intra-word amino-acid specificity is assessed using *Z*-score computation as described in Material and Methods. Briefly, we computed *Z*-scores for the 20 amino acids at the 7 positions of a structural word. We then considered the maximum *Z*-score, denoted *Z*_max_, measuring the strongest amino-acid specificity, and the number of significant positions, denoted nb_pos*_, indicating how many positions exhibit significant sequence specificity. As shown in Table 3, the global average *Z*_max _(resp. nb_pos*_) is equal to 10.3 (resp. 3.3). Almost every word (97%) present at least one significant position (Z_max _≥ 4) and 19% of words have at least one very significant position (Z_max _≥ 14). Conversely, only 3% of words (89 words covering 2% of loops) have no informative position. Among the sequence-informative words, 198 words (6% of recurrent words) are highly informative, as all their positions are significant. These very informative words cover 16% of loops. Words with high Z_max _contain structural letters D and S, in agreement with the fact that these two letters have very strong sequence specificity [51]. Thus we can conclude that most loops are composed of motifs with amino-acid specificities.

##### Correlation between structural variability and sequential specificity

**Figure 1:**
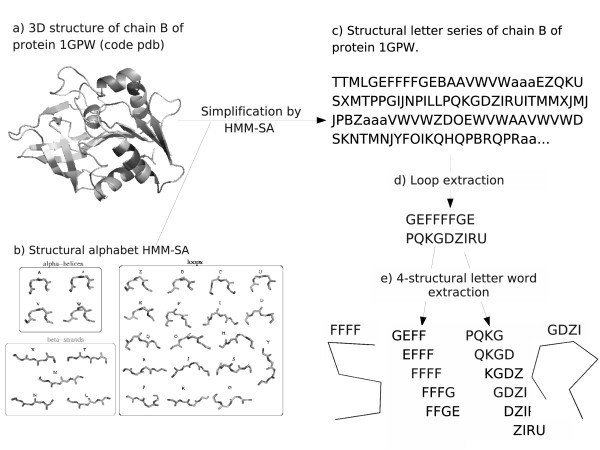
**Loop-word extraction from chain B of protein 1GPW**. a) 3D structure of the protein, b) the 27 structural letters of HMM-SA, c) structure simplification as a succession of structural letters, d) extraction of simplified loops, e) extraction of overlapping words of four structural-letters with structural words FFFF and GDZI illustrated.

We can note that there is no obvious link between Z_max _and RMSd_*w *_(Pearson coefficient is equal to 0.09, cf. Additional file 1). The structurally less variable words are not systematically the most informative ones in terms of amino acids. Some words with high RMSd_*w *_are informative in terms of sequence, as illustrated by word FFFF, with an RMSd_*w *_equal to 2.5 Å and *Z*_max _equal to 15.8 (an illustration of the word geometry is presented in Figure 1).

2590 words are characterized by both low structural variability and significant sequential specificity, with RMSd_*w *_lower than 1 Å and *Z*_max _greater than 4. These structural words cover 63% of loop regions. We can conclude that most loops are composed of motifs with a weak variability and amino-acid specificities.

#### Relation between structural words and loop type

After exploring the intrinsic structural and sequential properties of structural words, we analyzed their relationship with different loop types seen in proteins. We defined different loop-types according to their lengths and flanking secondary-structures [14,15,17,18].

##### Loop length

We used the Kullback-Leibler asymmetric divergence, denoted KLD criterion [52] (cf. Methods) to extract the words that are significantly more frequent in long loops than expected. These words are classified as specific to long loops. Words specific to short loops are extracted in a similar manner. The result of this analysis is presented in Table 4. We found that 758 words (23% of *W*set_≥30_) are specific to long loops and 476 words (14% of *W*set_≥30_) are specific to short loops. It means that roughly one third of the structural words display a significant preference for a length range, and two thirds are unspecific, i.e., shared by short and long loops. In Table 4, we also reported the loop coverage achieved by words specific to short and long loops. It can be seen that half of loops are covered by words shared by long and short loops. About one third of short loops (resp. long loops) are covered by words specific to short (resp. long) loops.

**Table 4:** Preference of structural words in *W*set_≥30 _according to the loop types as assessed by the KLD criterion and the associated loop coverage rate (on a per structural letter basis).

Loop words specificity	***W*set**_**≥**30_^**7**^	**UR**_***w***_^**7**^	**NS**_***w***_^**7**^	**OR**_***w***_^**7**^	**all loops**^**4**^	**short loops**^**5**^	**long loops**^**6**^
Long-loop-specific words	758 (22.9%)	23	475	260	23.2%	12.2%	33.9%
Short-loop-specific words	476 (14.4%)	23	220	233	25.7%	33.0 %	18.6%
Shared words 1	2076 (63.7%)	120	1519	437	45.9%	39.4%	56.3%
Flanking-region-specific words^2^	1879 (57.1%)	102	1131	646	58.6%	58.9%	58.4%
Flanking-region-unspecific words	1431 (43.2%)	64	1083	284	31.4%	21.7%	40.8%
Loop-type-specific words^3^	2543 (78.8%)	124	1605	814	66.3%	64.3%	68.2%
Loop-type-unspecific words	767 (23.2%)	42	609	116	16.6%	12.7%	20.5%

1: words shared by long and short loops 2: description by the four possible flanking-types (*αα*, *αβ*, *βα*, *ββ *loops), 3: description by the four flanking-types and the two length ranges (*αα*_*s*_, *αβ*_*s*_, *αβ*_*s*_, *ββ*_*s *_for short loops and *αα*_*l*_, *αβ*_*l*_, *αβ*_*l*_, *ββ*_*l *_for long loops). 4: all-loop coverage rate (on a per structural letter basis) 5: short-loop coverage rate (on a per structural letter basis) 6: long-loop coverage rate (on a per structural letter basis) 7: Number of words

##### Flanking regions

We now consider the four possible flanking regions for a loop: *ββ *: loops linking two *β*-strands, *αβ*: loops linking an *α*-helix and a *β*-strand, *αα *: loops linking two *α*-helices and *βα*: loops linking a *β*-strand and an *α*-helix. We found that about 60% of *W*set_≥30 _display a significant preference for one of the four-flanking-region types. This word set permits to cover about 59% of loops. Thus, about half of the loops are described by flanking-region-specific words.

##### Loop length × flanking regions

We then combine the loop length and loop type descriptors to distinguish eight types of loops. According to the KLD criterion, 2543 words (80% of *W*set_≥30_) exhibit a significant preference for one of the eight loop-types. This significant word set covers more than half of the loops (66%).

**Figure 2:**
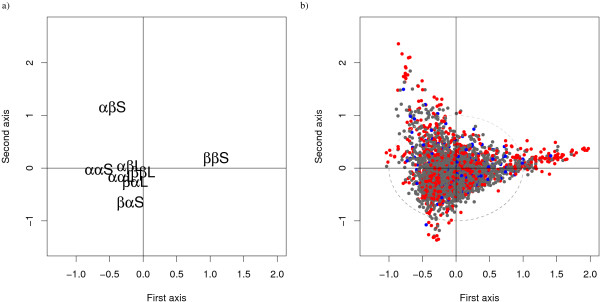
**Correspondence analysis between the eight loop-types (defined according the length and the flanking regions of the loops) and the structural words in *W*set_≥30_**. *αα*_*s*_, *αβ*_*s*_, *βα*_*s*_, *ββ*_*s *_correspond to the four different short-loop-types according to the flanking regions, and *αα*_*l*_, *αβ*_*l*_, *βα*_*l*_, *ββ*_*l *_correspond to the four different long-loop-types according to the flanking regions. *αα*: loops linking two *α*-helices, *αβ*: loops linking an *α*-helix and a *β*-strand, *βα*: loops linking a *β*-strand and an *α*-helix, *ββ*: loops linking two *β*-strands. The two first axes account for 36% + 26% = 62% of the variance. a) Plot of the eight loop types, b) Plot of *W*set_≥30 _words colored according to their statistical exceptionality: red = *OR*_*w*_, gray = *NS*_*w*_, blue = *UR*_*w*_.

The association between words and the eight loop types is further explored using a correspondence analysis presented in Figure 2. The first two axes of the correspondence analysis capture 62% of the variability and are mainly explained by the preference for short loops. The *ββ *short loops is opposite to the *αα *short loops on the first axis (36% of the variability) while the *αβ *short loop is opposite to the *βα *short loop on the second axis (26% of variability). Association is weaker for long loops -appearing in the central region of the plot- but similar tendencies are observed for short and long loops. This analysis made it possible to identify the loop structures with a dependence to loop-type, and the ones with no dependence.

#### Loop-type preferences × intrinsic properties

By combining the loop-type preferences of words and their intrinsic properties, we observe that words specific to short loops present slightly higher sequence dependence than others, while words specific to long loops have lower structural variability (cf. Table 5).

**Table 5:** Structural and sequential properties of loop-type specific words in *W*set_≥30_

Words characteristic	**shared words**^***a***^	short-long specific words	long-long specific words	flanking-region unspecific words	flanking-region specific words
Average RMSd_*w *_(Å)	0.90	0.80*	0.74	0.83	0.88
(± standard deviation)	(± 0.4)	(± 0.4)	(± 0.4)	(± 0.4)	(± 0.4)
AverageZ_max_	9.4	15*	9.7	8.5	11.6*
(± standard deviation)	(± 4.7)	(± 9.5)	(± 5.3)	(± 3.9)	(± 7.0)
Average nb_pos*_	3.0	4.6*	3.3	2.7	3.8
(± standard deviation)	(± 1.6)	(± 1.7)	(± 1.8)	(± 1.5)	(± 1.8)

The upper part of the table corresponds to the analysis of word structural properties. The intra-word structural variability is analysed using the Root Mean Square deviation (RMSd) between fragments corresponding to the same word (RMSd_*w*_). The inter-word structural variability is analysed using the RMSd between fragments of two different words (RMSd_*dev*_). The lower part of the table corresponds to the analysis of sequential properties of words. The intra-word amino-acid preferences of a word are analysed using *Z*_max _criterion (cf. Method section) and the number of significant position of a word (nb_pos*_). Numbers within brackets indicate standard deviations. *: significant differences according to the Kruskal-Wallis test. The RMSd_*dev *_are computed on a subset of 890 words of *W*set_≥30_. ^*a*^: words sharing by long and short loops

We can note that only 44 words (1% of the *W*set_≥30 _words) have neither amino-acid-significant position, nor loop-type preference. Thus, less than 1% of loop regions are covered by these unspecific words in terms of sequence dependence and loop types.

Our approach, which relies on a systematic decomposition of short and long loops, allowed showing loops are composed of recurrent structural motifs, some of them with preference for a particular loop type in terms of loop length and/or flanking regions. Conversely, some structural words have no preference for a loop length, meaning that they are similarly found in short and long loops.

### Second part: Statistical exceptionality of structural words

In the second part of this study, we complement our analysis of word properties by their statistical exceptionality in protein structures represented by strings of structural letters. Statistical exceptionality is traditionally used in genome analysis to extract functional motifs such as enzyme restriction sites or regulatory motifs [44-48]. Our goal was to explore if a statistical bias is also associated to specific properties in the case of protein structures. Statistical exceptionality does not measure the frequency of a word. It is an indicator of the discrepancy between observed and expected occurrence according to a background model that takes into account the first order Markovian process between structural letters. The statistical representation of words was assessed using the SPatt software that computes an exceptionality score *L*_*p *_for each word (see Material and Methods). According to the value of *L*_*p*_, words are classified as over-represented, under-represented or not significant. Hereafter, over-represented words are referred to *OR*_*w*_, under-represented words as *UR*_*w *_and not significant words as *NS*_*w*_.

#### Extraction of exceptional words

The analysis of the correlation between the frequencies (i.e. cluster size) and *L*_*p *_values for all words in the data set shows that many frequent words tend to be over-represented but there is no linear relation between frequency and exceptionality (cf. Additional file 1). Some frequent words are classified as *UR*_*w *_or *NS*_*w*_, like FFFF (seen 537 times, *L*_*p *_= -2.8). Conversely, some rare words are classified as *OR*_*w*_, like GDZI (seen 64 times, *L*_*p *_= 102.2). An illustration of the geometry of these words is presented in Figure 1. This result shows the relevance of the extraction of word exceptionality instead of word frequency.

The repartition of words in *W*set_≥30 _according to exceptionality status is given in Table 1. We can see that *OR*_*w *_contribute predominantly to the set of fragments in *W*set_≥30_: 40% of the fragments are in *OR*_*w *_clusters. *OR*_*w *_clusters are indeed significantly bigger than other word types (cf. Table 1).

#### Redundancy of loops and robustness of the extraction method

In this study, loops were extracted from a non-redundant data set presenting less than 50% sequence identity. Different redundancy levels have been used in the literature. Concerning loop classifications, Wloop[16] used a protein data bank with 50% sequence identity. The loop classification system ArchDB is available in two versions: one built on a set of proteins with 40% sequence identity and the second on a redundant-protein set with 95% sequence identity [18]. It is classically considered that the evolutionary relationship between two proteins is detectable up to 25% sequence identity. Consequently this cut-off is frequently used for calibrating prediction methods [53]. Since loops are more variable than the rest of the protein sequence, we set the identity cut-off at 50% in order to work with as many data as possible with limited redundancy.

One could object that no attention was given to how many redundant loops were left or removed from the database during the redundancy filtering. The problem of loop redundancy is a non-trivial one: the extraction of loops from a non-redundant protein set does not necessarily result in a non-redundant loop set, and loop redundancy is itself difficult to quantify. We indirectly addressed this question by repeating our systematic extraction on different data sets, using identity levels of 25% and 80%. It was also important to ensure that our observations were applicable to protein structures in general and not only to the data set used. Taking into account the correction due to the different database sizes (see Method), we found a satisfactory level of consensus equal to 82% between the 25% and 50% databases, and 90% between the 50% and the 80% databases (more details are given in Additional file 1). These ratios refer to the proportion of recurrent words - common to both data sets - that are classified in the same statistical word type (over-presented/not significant/under-represented). Moreover, only one word, QLHB, was assigned as over-represented in a data set and under-represented in the other. Therefore, we can conclude that the extraction of exceptional words is robust and very weakly depends on the redundancy of the data set. Then, we compared the properties of the *W*set_≥30 _words after classification into these three classes.

#### Exceptionality and word properties

The structural and amino-acid property measures for the three statistical word types (*OR*_*w*_, *NS*_*w *_and *UR*_*w*_) are reported in Table 3.

**Figure 3:**
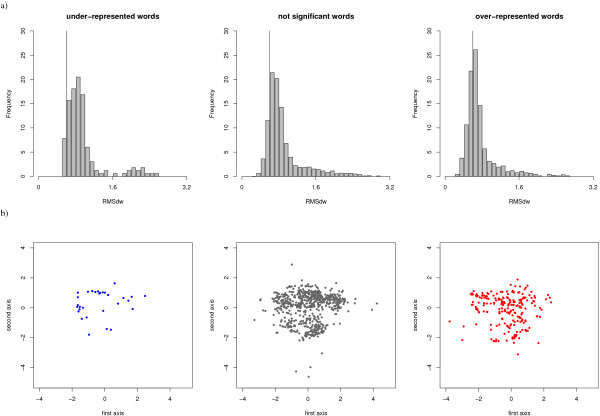
**Structural variability of the three statistical word types of *W*set_≥30_**. a) Intra-word structural variability: distribution of the RMSd_*w*_. The vertical line corresponds to a threshold of 0.6 Å b) Inter-word structural variability: Sammon's map computed from the RMSd_*dev *_for a sample of 890 words of *W*set_≥30_. All the points are subjected to the same projection and plotted on distinct plots.

**The intra-word structural variability **is lower for *OR*_*w *_than for other words, as assessed by a Kruskal-Wallis test [54] (p-value < 2 × 10^-16^, cf. Table 3). The RMSd_*w *_distribution for the three statistical word types is shown in Figure 3a. It can be seen that the RMSd_*w *_distribution of *OR*_*w *_is shifted toward lower values. *OR*_*w *_are thus significantly less structurally variable than other words.

**The coverage of the structural space **by the structural words of different exceptionality status is assessed by the RMSd between clusters. The goal is to evaluate how well the structural words sample the conformational space of loops. In order to assess the coverage of the loop-conformational space, we computed the RMSd between all pairs of words in the *W*set_≥30_, denoted RMSd_*dev*_. The average RMSd_*dev *_computed for each type of words is given in Table 3. The average RMSd_*dev *_for words in *W*set_≥30 _is equal to 2.7 Å It is significantly greater than the average RMSd_*w*_, indicating that the structural variability of words is low compared to the structural differences between words. This observation stands for the three types of words. RMSd_*dev *_were computed between every words of *W*set_≥30_, and the resulting 3310 × 3310 dissimilarity matrix is used to compute Sammon's map projections shown in Figure 3b. It can be seen that the three statistical word types all sample the conformational space in the same way. It means that *OR*_*w *_correctly sample the *W*set_≥30 _conformational space and are not restricted to some particular shapes. Let us note that RMSd are dissimilarity measures that do not necessarily respect the triangular inequality. A consequence is that the Sammon's projection does not actually reflect the word's proximity (words separated on the map can be structurally close). However, since the three point series are simultaneously projected on the same subspace, Sammon's maps can be used to qualitatively assess the similarity between the conformational sampling. We can thus conclude that *OR*_*w *_are, on average, significantly more structurally stable than other words, and sample all the conformational space.

**Figure 4:**
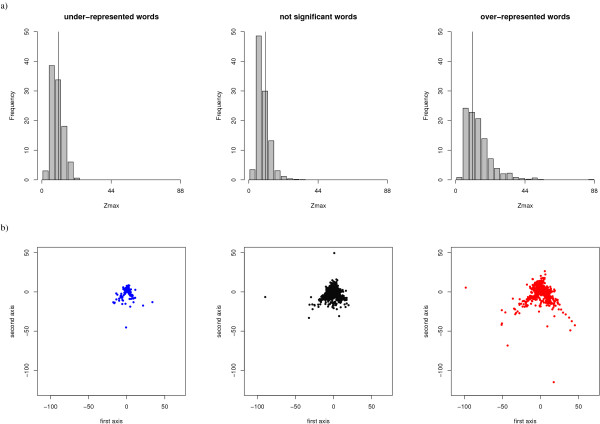
**Sequential specificity of the three statistical word types of *W*set_≥30_**. a) Intra-word analysis: distribution of the Z_max_. The vertical line corresponds to a threshold of 10. b) Inter-word analysis: Sammon's map computed from the Euclidean distance between *Z*-scores. All the points are subjected to the same projection and plotted on distinct plots.

**Intra-word amino-acid specificity **is significantly higher for *OR*_*w *_(p-value < 2 × 10^-16^, cf. Table 3). The Z_max _distributions for the three statistical word types are shown in Figure 4a. The distribution for *OR*_*w *_is clearly shifted toward high values of Z_max_. *OR*_*w *_are also more informative in terms of number of significant positions (p-value < 2 × 10^-16^, cf. Table 3). These results must be interpreted with caution due to the restrictive condition for the interpretation of the Z-scores (see Material and Methods). However, they show that *OR*_*w *_are, on average, more informative in terms of both the number of significant positions and specificity.

**The coverage of sequence space **by the different structural words is assessed using a procedure similar to the one used for structural space. We computed the Euclidean distances between Z-score vectors of each word pair in *W*set_≥30_. The resulting average distances are given in Table 3. The Kruskal-Wallis test indicates that, in terms of amino-acid specificity, *OR*_*w *_are significantly more distant one from the other (p-value <2.2^-16^, cf Table 3). Sammon's map projections of the three word-types are shown in Figure 4b. We can see that *OR*_*w *_cover a large region of the map, including regions not visited by *NS*_*w *_and *UR*_*w*_. We can conclude that *OR*_*w *_are globally more distinct from each other in terms of amino-acid sequence dependence than other words and that they sample the sequence space better than other word types.

#### Exceptionality and loop types

As shown in Table 1, *OR*_*w *_significantly contribute to the description of long loops: *OR*_*w *_cover about 40% of both short and long loops. Moreover, 58% of the loops contain at least one *OR*_*w*_, and as many as 80% of long loops contain at least one *OR*_*w*_. If we consider the specificity of words for a particular loop length (cf. Table 4) it can be seen that 260 *OR*_*w *_are specific to long loops and 233 *OR*_*w *_are specific to short loops. It means that 493 *OR*_*w *_out of 930, i.e. 53% of *OR*_*w*_, exhibit a significant preference for a loop-length type. This proportion should be compared to what is obtained for other words: 31% of *NS*_*w *_and 28% of *UR*_*w *_are significantly dependent on a particular loop length range. If we consider the flanking secondary-xstructures, the same observation can be made: 70% of *OR*_*w *_versus 52% of *NS*_*w *_and 45% of *UR*_*w *_are specific to a particular loop type. It thus seems that *OR*_*w *_exhibit stronger dependence toward the loop type than other statistical word-types.

Finally, we compared the preference of the three word-types for the eight loop-types defined by length range and flanking secondary-structures. We found that 88% of *OR*_*w *_versus 72% of *NS*_*w *_and 75% of *UR*_*w *_exhibit a significant dependence for a particular loop type. The qualitative analysis by correspondence analysis is displayed in Figure 2, where the three statistical word types are highlighted in different colors. It can be seen that *OR*_*w *_predominantly appear in outlying regions of the plot, in agreement with the KLD quantification.

Therefore, we can conclude that *OR*_*w *_present higher signature in terms of structure and/or sequence and higher dependence to loop types than other words. At the same time, *OR*_*w *_correctly sample all the loop-conformational space, and better cover the sequential space of protein loops. They are seen in every loop type and offer a reasonable coverage rate, with only 930 different structural motifs.

## Discussion and Conclusion

In this study, we have developed an original approach for the analysis and the description of loop structures. This approach corresponds to a systematic extraction and statistical analysis of seven-residue structural motifs within loops, using a structural-alphabet simplification. Contrary to classic approaches, our method does not require either loop-structural alignment or computation of structural parameters. The structural word approach defines a structure-based clustering of all fragments, where all seven-residue fragments encoded in a similar word can be seen as a cluster. Our systematic clustering resulted in 28274 clusters, with 1 to 1633 fragments per cluster, and an average size equal to 15. The analysis of B-factors showed that some of the singletons are indeed associated to regions with high B-factors, which is indicative of coordinate uncertainty. It was thus legitimate to exclude them from the analysis.

In order to compute cluster properties, we chose to restrict ourselves to the 3310 clusters (= 12% of clusters) with more than 30 fragments, referred to *W*set_≥30_. This reduction was required to have a sufficient number of fragments to compute RMSd and sequence profiles for clusters. This limited number of structural words (3310) results in a good coverage rate of the loops: 73% of loop-lengths. We additionally checked that the restriction to *W*set_≥30 _does not result in the restriction to highly populated structural families, and that our results are stable on different data sets.

### Comparison with existing approaches

An extensive comparison with already existing loop classification schemes is extremely difficult because we do not consider the same objects, and pursue different objectives. Existing classifications cluster loops according to their length [12,14,15,17,18], flanking region types [12,14,17,18,20,21], flanking region geometry [12,14,17,18] and loop geometry [17,18]. Such classifications consider full length loops and are thus inherently limited to short loops. In the present study, we cluster fixed-length structural motifs within loops, independently of their lengths or flanking regions, thus also bringing information for long loops. Consequently, it is delicate to compare our loop analysis with existing loop classifications.

Other studies have previously investigated the use of seven-residue fragments to analyze protein structures [55,56] whereas our study focuses on loop structural fragments. For this reason, the results are not directly comparable.

Other studies consisted in identifying functional patterns in whole proteins [57,58]. Such patterns, involved in protein function, are relatively rare. On the contrary, our approach considers recurrent structural motifs in loops. Alternatively, some groups have investigated the identification of 3D structural patterns linked to functions that are not necessarily made of consequent residues [59-63]. For example, Ausiello et al. (2009) [63] extracted some structural motifs from protein in different folds which recognize ligands presenting same features. In this case also, the studied objects are very different, making the comparison difficult. Another interesting analysis, MegaMotifBase, deals with structural motifs that are important for the preservation of the 3D structure in given families or superfamilies [64]. These motifs were identified using both sequence conservation and preservation of important structural features. They mainly correspond to regular secondary structures, whereas we focused our analysis on loops. For all these reasons, any comparison between our approach and already existing classifications should be regarded with caution.

### Insight into loop structures

We analyzed structural and amino-acid properties of clusters, defined by structural words, using RMSd and different criteria to measure their amino-acid dependencies. We found an average intra-cluster RMSd_*w *_equal to 0.85 Å versus 2.72 Å for the inter-cluster RMSd_*dev*_, which confirms our previous results [40]. In the loop classification ArchDB[65] clusters grouping seven-residue loops present an average RMSd close to 1 Å. In Sander et al. [55], fragments were clustered according both to their structure and amino-acid sequence into 27 clusters with an average RMSd of 1.19Å. The most populated cluster groups *α*-helix fragments and probably largely contribute to the average RMSd.

Loop description by recurrent structural words permits a quantification of the loop structural redundancy: around 73% of loops are described by a limited number of accurate recurrent structural words. Thanks to the loop-structure simplification using HMM-SA, our method is the first one allowing a systematic mining of loops independently of their lengths and the study of all loops in terms of motif composition.

First, we demonstrate that the majority of the recurrent structural words have low structural variability and specific sequence signature. The simplification of loop structures using HMM-SA permits to analyze long loops. We can observe that 46% of loops are covered by words found both in short and long loops. These results show that short and long loops are composed of similar motifs. This is in agreement with the insertion/deletion process of loop evolution hypothesis made in [66]. In addition to the identification of the shared structures, our analysis provides a quantification of how the same structural words are re-used in different loops. The existence of words found in both long and short loops could allow transposing some short-loop results into the long-loop analysis and decreasing the long-loop-analysis complexity.

We observe that only one third of short (resp. long) loops are covered by words that are specific to short (resp. long) loops. Moreover, words specific to short loops have higher amino-acid specificities than other words. That means that these short loop regions (30% of short loops) are more informative in terms of sequence than other regions. Interestingly, words that are specific to long loops are structurally less variable than others meaning that a part of long loops (34%) are structurally well defined.

We also analyze the dependence between recurrent words and the loop flanking-regions. We show that around 60% of words exhibit a significant preference. Most of these words are specific to *βα *and *ββ *loops. These results are in agreement with classification of short loops based on flanking region information as [12,14,15,17,18,20,21] and provide an identification and quantification of the structures with a dependence on the flanking regions. Moreover, this study allows identifying and quantifying regions with no preference for flanking-region types. Indeed, 31% of loops are covered by words with no preference for a flanking-region type.

The amino-acid specificities of structural words were also assessed. We observed that 97% of recurrent words, covering 70% of loops, have amino-acid specificities. Different studies have analyzed the amino-acid preferences of loops, particularly for short loops. Kwasigroch et al. (1997) have shown that amino-acid preferences were more frequent in the core of short loops [15,16]. Other studies have focused on the amino-acid preferences of *β*-turns and shown that these amino-acid preferences occurred at end positions [25,67]. This study provides an identification of regions with amino-acid specificities and a new quantification of the amino-acid specificity: we found an average number of three positions with significant amino-acid preference for *W*set_≥30 _motifs.

### Perspective in terms of loop-structure prediction

Most recurrent motifs exhibit significant amino-acid specificities: half of them display significant level of amino-acid conservation in at least four significant-positions. If we consider words with at least four significant-positions as predictable, we extract 1359 words covering 60% of the loops (on a per-structural letter basis). It is clear that this predictability index (at least four significant positions) is very basic and too optimistic. The predictability index of a word has to combine both its sequence informativity and sequence specificity. Indeed, one word can have several positions with high amino-acid preferences but close sequence from other words. Conversely, words with few informative positions can be clearly distinguishable from others in terms of sequence. Moreover, several words can be compatible with a same seven-residue sequence, involving several candidates per amino-acid sequence. A possible strategy for loop prediction would consist in splitting the query sequence into overlapping seven-residue fragments, and identifying subset of structural words compatible in terms of sequence profile with each fragment.

The successions of compatible overlapping word candidates would then be selected using a hidden Markov model taking into account the favorable transitions between structural words. This would result in a 1D structural letter trajectory set compatible with the target loop sequence. Then, the 3D reconstruction from this set of 1D trajectories could be achieved using an energy function as in PEPfold [68]. This approach could yield a set of 3D structural conformation candidates for the target loop, in agreement with the flexibility of loops. Finally, for long loop prediction, a confidence index could be proposed for different parts of the predicted loop. Indeed, for a given loop, prediction of some regions could result in a limited number of word candidates while for other regions, the prediction could result in a large number of word candidates. This approach could be a way to decrease the complexity of long-loop prediction.

### Illustrative Example of loop analysis

**Figure 5:**
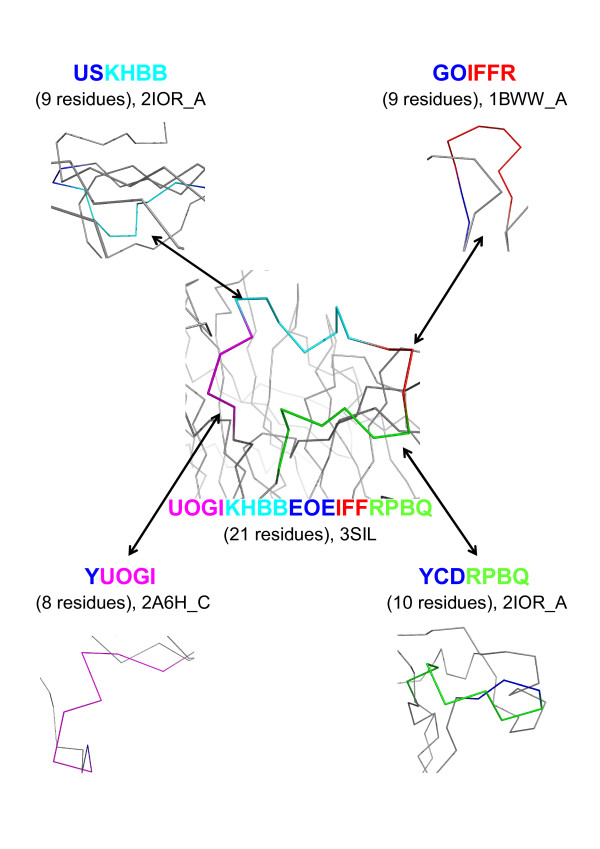
**Recurrent words found both in long and short loops**. A long loop of 18 structural letters (central figure) extracted from protein with pdb code 3SIL contains four words (UOGI, KHBB, IFFR, RPBQ) of *W*set_≥30_. The protein is colored in gray, and the loop in blue except the four words UOGI in magenta, KHBB in cyan, IFFR in red and RPBQ in green. These four words are also seen in short loops in other structures. For each word, we indicate the structural letter pattern, the loop length within brackets and the pdb code of the protein structures. Structures are displayed with pymol [78].

In Figure 5, we present an illustration of a long loop of 18 structural letters extracted from the protein structure with pdb code 3SIL, encompassing residues 120 to 140. Using the word extraction protocol, this loop was decomposed into 15 words of 4 structural letters. Among these 15 words, four words -namely UOGI, KHBB, IFFR and RPBQ- belong to *W*set_≥30_. These four words are seen in both short and long loops in the data set, as illustrated in Figure 5. Structural word KHBB is over-represented, with an *L*_*p *_value equal to 39.5. It is characterized by a low structural variability (RMSd_*w *_= 0.4 Å) and strong amino-acid preference (*Z*_*max *_= 25), with conservation of hydrophobic amino acid at position 2 and Proline at position 3. These amino-acid conservation trends are derived from the analysis of every occurrence of a particular fragment.

**Figure 6:**
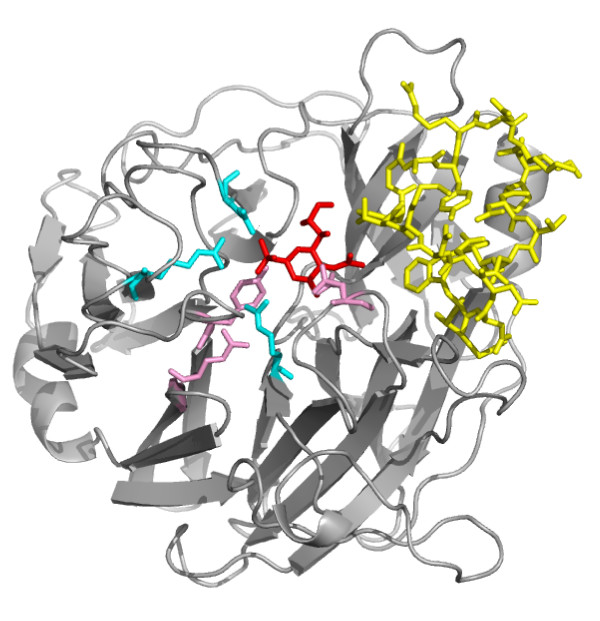
**Functional residues of sialidase **3SIL. Catalytic and binding residues annotated in Swiss-Prot are highlighted in pink and cyan. The inhibitor (found in structure 1DIL) is highlighted in red. The long loop revealed by the structural word analysis is highlighted in yellow.

In this particular protein, a Lysine and a Threonine occupy positions 2 and 3 of word KHBB. This region does not appear to be particularly conserved in the multiple alignment of homologous sequences retrieved from a BLAST search in Swiss-Prot (data not shown). When aligned with sequences retrieved from a BLAST search in PDB sequences, this region exhibits three positions with equivalent residues (see alignment in Additional file 1). We attempted to further explore the functional implication of this long loop. 3SIL is a sialidase from *Salmonella typhimurium*. It corresponds to Swiss-Prot entry NANH_SALTY, and is responsible for the cleavage of terminal sialic acid from glycoproteins. There is no functional annotation in Swiss-Prot for the 120-140 region, but the catalytic and substrate-binding sites are annotated. They are highlighted in pink and blue in Figure 6. Furthermore, a structure of sialidase co-crystallized with an inhibitor is available in the PDB: structure 1DIL, with sequence identical to 3SIL. The inhibitor is thus shown in red in Figure 6. It can be seen that loop 120-140 is spatially close to functional residues and inhibitor molecules. This observation suggests that this loop could be important for the substrate stabilization, but only the observation of the enzyme co-crystallized with a substrate could confirm this hypothesis.

This example shows that some motifs extracted from loops seem to be involved in protein function. It is not surprising due to the fact loops are often involved in protein function.

### Perspective of functional-motif identification

In genomic sequences, functional motifs are often characterized by particular frequencies (rare or very frequent). Therefore, the search for functional motifs is successfully guided by the search for exceptional motifs [44,45]. Inspired by this singularity, we explored the properties of structural words in proteins to see if the over- or under-representation of particular conformations can be linked to particular features. Contrary to classic methods that were primarily developed for DNA sequences, statistics are here computed by a method that takes into account the large number and short length of sequences of our data set [69]. We considered the intrinsic properties of structural words and their relationship with the statistical exceptionality status of words, classified as over-represented, under-represented, or not significant. The comparison of the three statistical word types showed that over-represented words have indeed specific properties: they are highly conserved in terms of structure or sequence and highly dependent on loop types. By setting a RMSd_*w *_cut-off equal to 0.74 Å and a Z-max cut-off equal to 14, we found that 89% of over-represented words present either a low RMSd or a high Z_max _or a significant dependence to a loop type defined by eight types according to the KLD criterion. This ratio is only 62% for other words. This indicates that statistical exceptionality results from a complex process combining word frequency, sequence and/or structure properties. The consideration of statistical exceptionality thus enhances the signal-to-noise ratio in protein loops. Most of the time, the relationship between local structures and protein function is not straightforward. Our findings open new perspectives to the use of over-representation in order to detect functional motifs in loops. It is the subject of an ongoing study (Regad et al, in preparation) where we suppose that functional motifs could correspond to over-represented motifs in a protein family.

## Methods

### Data

We used a data set of protein structures corresponding to chains presenting less than 50% of sequence pairwise identity extracted from PDB of May 2008. The data set is composed of 8186 protein chains of at least 30 residues, obtained by X-ray diffraction with a resolution better than 2.5 Å. Proteins for with missing residues or alternate conformations were removed.

### Structure simplification using HMM-SA

Our structural alphabet, HMM-SA, is a library of 27 structural prototypes of four residues, called structural letters, established using a hidden Markov model [42,70]. Thanks to HMM-SA, the 3D structure of a protein backbone is simplified into a sequence of structural letters. The simplification relies on C*α *positions only: each four-residue fragment of the protein structure is described by four inter-C*α *distances. Consecutive four-residue fragments are overlapping on three residues resulting in one common distance. The resulting distances are the input of a hidden Markov model, and the 3D structure is translated as a sequence of 1D structural letters. This translation is made using the Viterbi algorithm [71] and takes into account both the structural similarity of the fragments with the 27 structural letters of the structural alphabet and the preferred transitions between structural letters. A protein structure of n residues is then simplified as a sequence of (*n *- 3) structural letters. The 27 structural letters, named [A-Z, a] are shown in Figure 1. It has been shown previously [51], that four structural-letters, [a, A, V, W], specifically describe *α*-helices, and five structural letters, [L, M, N, T, X], specifically describe *β*-strands. The remaining 18 structural letters [B, C, D, E, F, G, H, I, J, K, O, P, Q, R, S, U, Y, Z] allow accurately describing loops. Some transitions between structural letters are not possible, which results in a limited number of pathways between letters and in a limited number of short patterns of structural letters.

### Extraction of structural motifs within loops

Following our previous study [40], loops are identified as series of structural letters linking simplified regular secondary structures (*α*-helices and *β*-stands) that are defined using regular expressions of structural letters. This approach permits to extract a bank of 93396 simplified loops ranging from 4 to 82 structural letters with an average length of 8.5 ± 5.5 structural letters, corresponding to an average length of 11.5 ± 8.6 residues. A loop of *l *structural letters corresponds to (*l *+ 3) residues. Long loops -more than 12 residues- represent 28% of the loops in our data set. 39% of the loops are linking two *β*-strands, 23% are linking a *β*-strand to an *α*-helix, 22% an a-helix to a *β*-strand, and 16% two *α*-helices. The extraction of structural motifs in loops is illustrated in Figure 1. Simplified loops are split into series of overlapping words of four structural-letters, i.e., seven residues. A loop of *l *structural letters is then split into (*l *- 3) words. As we focus on structural motifs within loops, words beginning or ending with a structural letter specific to regular secondary structures [AaVWLMNTX] are excluded. This results in a global set of 28274 structural words describing all loops in the simplified structural alphabet space. The structural words thus define a partition of the structural diversity of loops, where each four-structural-letter word is a cluster of seven-residue fragments.

### Loop coverage by structural words

The coverage rate of loops by a word set corresponds to the percentage of loop structural-letters covered by these words.

For example, given two loops of 11 (*l*_11_) and 15 (*l*_15_) structural letters and a set of recurrent 4-structural-letter words (*S*_*w*_). Loop *l*_11 _contains two words of *S*_*w *_on positions 1 to 4 and 8 to 11. As these two words are not overlapping, they cover 8 structural letters. Loop *l*_15 _contains three words of *S*_*w *_on positions 1 to 4, 3 to 6 and 9 to 12. As the first two words are overlapping, these three words cover 10 structural letters. Thus, the coverage rate of these two loops by *S*_*w *_is equal to  = 69%.

This coverage rate is used in order to provide information on loop description by a set of structural words.

### Structural variability of words

#### Intra-word

The structural variability of a structural word is measured by the geometric variability of the seven-residue fragments encoded by that word, computed using *C*_*α *_Root-Mean-Square deviation (RMSd_*w*_). It is obtained by computing the average RMSd_*w *_between 30 randomly selected fragments in the cluster. It is only computed for words seen more than 30 times.

#### Inter-word

The structural dissimilarity between two words is similarly measured by the average C_*α *_Root-Mean-Square deviation (RMSd_*dev*_) between 30 fragment pairs randomly selected within pairs of seven-residue fragments encoded by the two words. The word-structure-space coverage is analyzed by a Sammon's map [72] performed using the C_*α *_RMSd_*dev *_dissimilarity matrix

### Sequential specificity of words

Although the structural-alphabet decomposition into structural word is purely geometrical, it is still possible to analyse the sequence-to-structure dependence *a posteriori*. This is achieved using *Z*-score computation.

#### Intra-word

For a word *w*, we compute a *Z*-score for each of the 20 amino acids at each of the 7 positions of fragments corresponding to the word.

The *Z*-score of amino acid a, (1 ≤ *a *≤ 20) at position l (1 ≤ ℓ = 7) of a word *w*, is obtained by comparing the observed frequency of amino acid *a *at position ℓ in word *w *with its expected one:(1)

To facilitate the computation of Z-scores, we approximate the distribution of amino acid *a *in position ℓ of word *w *(corresponding to a binomial distribution ℬ(*N*_*a*,ℓ_, )) by a Poisson distribution (*N*_*a*,ℓ_·*N*_*w*_), Where(2)

where *N*_*w *_is the frequency of *w *and *N *is the total number of words in the whole data set.

To analyze the significance of a *Z*-score, the expected frequency (*N*_*a*,ℓ, *w*_) must be greater than 5. A positive Z-score corresponds to an over-representation of the amino acid, and a negative one corresponds to an under-representation of the amino acid.

A word is thus described by a vector of 140 (7 positions × 20 amino acids) Z-scores. From these 140 Z-scores, two criteria are used to assess the amino-acid informativity of each word. The first criterion, denoted Z_max_, corresponds to the maximum *Z*-score among the 140. It measures the strongest amino-acid specificity among the 7 positions of a word. The second criterion, named nb_pos*_, 1 ≤ nb_pos* _≤ 7, corresponds to the number of positions of word *w *where at least one amino acid is significant in terms of *Z*-scores. Significance cut-off is set to 4 using Bonferroni correction. It should be noted that this second criterion underestimates the sequence informativity because of the limitation introduced by the *Z*-score validity condition (only *Z*-scores with expected frequency (*N*_*a*,ℓ, *w*_) higher than 5 can be considered for significance).

#### Inter-word

To check if two words have close amino-acid-sequence preferences, the Euclidean distance between their 140 *Z*-score vectors is computed [73]. The coverage of sequence specificity of words is analyzed by a Sammon's map performed using this Euclidean distance [72].

### Loop type specificity of words

To study the preference of structural words for particular ℓ loop types (defined by length and/or flanking regions with ℓ, 1 ≤ ℓ ≤ *N*_ℓ_) the word distribution in different loop types is compared to the global distribution of loop types using a relative entropy measure, called the Kullback-Leibler asymmetric divergence, Kullback distance or relative entropy, denoted KLD [52]. The KLD quantifies the preference of a word w for the loop types, as:(3)

where *p*_*w*, ℓ_, denotes the relative frequency of word *w *in loop type ℓ and *p*_ℓ_, the relative frequency of loop type ℓ among all loops. The KLD is equal to 0 if is *w *is similarly distributed in every loop type and increases with loop type dependence. The significance of KLD value is assessed by a chi-square test, since the quantity 2 × *N*_*w *_× *KLD*(*w*) follows a chi-square with *N*_*w *_- 1 degrees of freedom. Thus, words associated to specific loop types have significant KLD values. A correction is introduced using False Positive Rate (FPR) to take into account multiple testing. A correspondence analysis is used to visualize the main relationships between words and loop types.

### Loop-word statistical exceptionality

The principle is to compare the actual frequency of a word in the data set and its expected frequency under a background reference model. A word seen significantly more (respectively less) than expected is then classified as over-represented (respectively under-represented). The expected frequency is computed using a Markov model for which the parameters are estimated from the global set of loops. This is performed using the software SPatt [74] available at http://stat.genopole.cnrs.fr/spatt, with a first order Markov chain used as reference. SPatt approach is based on the Pattern Markov Chain (PMC) notion [75]. This software has been adapted to the case of data sets with a large number of short sequences [43]. The statistical significance of the exceptionality is quantified by a p-value. To facilitate the analysis, p-values are translated into scores using equations:(4)

where *N*(*w*) is the expected frequency of the word *w*, and *N*_*w *_its observed frequency. An over-represented word has a positive *L*_*p *_value and an under-represented word has a negative *L*_*p *_value. For example, an *L*_*p *_equal to 21.3 means that the word is over-represented with a p-value equal to 10^-21.3^. A *L*_*p *_equal to -17.7 means that the word is under-represented with a p-value equal to 10^-17.7^. The *L*_*p *_threshold for statistical significance is set to 5.94, using the Bonferroni adjustment to take into account multiple tests. This permits to classify words as over-represented (*L*_*p *_> 5.94), under-represented (*L*_*p *_< -5.94) or not significant (-5.94 ≤ *L*_*p *_≤ 5.94).

As explained in [75], pattern significance scores tend to increase with the considered database size. This is due to the fact that a tail distribution event like the one we usually consider in pattern problems (i.e. pattern with small p-value) falls within the range of the Large Deviations theory [76,77] which means that its probability p to occur can be approximated by *p *≃ exp(-ℓ*I*) where *I *is a real positive rate and ℓ is the database size. As a consequence we have log *p *≃ -ℓ*I *which is exactly the pattern score we consider (up to a constant multiplier). It is hence obvious that extreme pattern scores will increase in magnitude linearly with database size. If this is not a problem when we perform a pattern analysis on a single database, this bias has obviously to be corrected in order to compare results from two different databases. The correction simply consists in using one of the database as a reference and rescaling the pattern scores obtained on the second database by the appropriate ratio of sizes.

## Authors' contributions

LR, JM, and ACC conceptualized the project. LR developed the software, performed the experiments and drafted the paper. NG developed and adapted the software SPatt. LR, JM and ACC analyzed the experimental results. LR, JM and ACC contributed to writing the paper. All authors read and approved the final manuscript.

## Supplementary Material

Additional file 1 **Supplementary**. This file is a pdf file. It contains different information about: • Extraction of words of different lengths. • Comparison of the loop length distribution in loops containing all words and loops containing only words seen 30 times. • Coverage of SCOP superfamilies by recurrent words. • Correlation between sequence specificity (*Z*_max_) and structure variability (RMSd_*w*_) for all words in *W*set_≥30_. • Exceptionality score *L*_*p *_versus frequency for the 28274 words of the data set. • Robustness of the word statistical analysis on different data sets. • ClustalW of 3SIL sequence (P29768) and homologous sequences from UniProt Click here for file
