# Supplementary material for: Mining protein loops using a structural alphabet and statistical exceptionality
Source: BMC Bioinformatics. 2010 Feb 4;11:75. doi: 10.1186/1471-2105-11-75 (PMC2833150; doi:10.1186/1471-2105-11-75)
Supplement: Additional file 1 — Supplementary. This file is a pdf file. It contains different information about: • Extraction of words of different lengths. • Comparison of the loop length distribution in loops containing all words and loops containing only words seen 30 times. • Coverage of SCOP superfamilies by recurrent words. • Correlation between sequence specificity (Zmax) and structure variability (RMSdw) for all words in Wset≥30. • Exceptionality score Lp versus frequency for the 28274 words of the data set. • Robustness of the word statistical analysis on different data sets. • ClustalW of 3SIL sequence (P29768) and homologous sequences from UniProt [file 1471-2105-11-75-S1.PDF]

Additional file 1

Mining protein loops using a structural alphabet and statistical exceptionality

Leslie Regad, Juliette Martin, Grégory Nuel, and Anne-Claude Camproux.

1- Extraction of words of different lengths

1.1- Table of the word extraction

Word length (structural letters)		2	3	4	5	6	7
Fragment length (residues)		5	6	7	8	9	10
Number of different words		213	3014	28274	113766	189846	199559
Number of fragments		564321	489308	415071	338874	277509	228227
Mean occurrence		2649.36	162.35	14.68	2.98	1.46	1.14
Standard deviation		2810.81	293.07	35.98	6.49	1.89	0.82
Occurrence min		11	1	1	1	1	1
Occurrence max		14920	3577	1633	850	200	104
Occurrence Interval comprising 80% of words	Quantile 10%	248	3	1	1	1	1
	Quantile 90%	5603.6	386	34	6	2	1
	Number of words corresponding to 80%	170	2411	22619	91012	151877	159647
	Number of fragments corresponding to 80%	451457	391446	332057	271099	222007	182582

Table 1: Statistics of occurrence of words of 2, 3, 4, 6, and 7 structural letters, corresponding to fragments of 5, 6, 7, 8, 9, and 10 residues

1.2- Distribution of word occurrences

To choose the optimal length of word, we computed the frequency of all structural words in our data set, with length from 5 residues (2-structural letters) to 10 residues (7-structural letters).

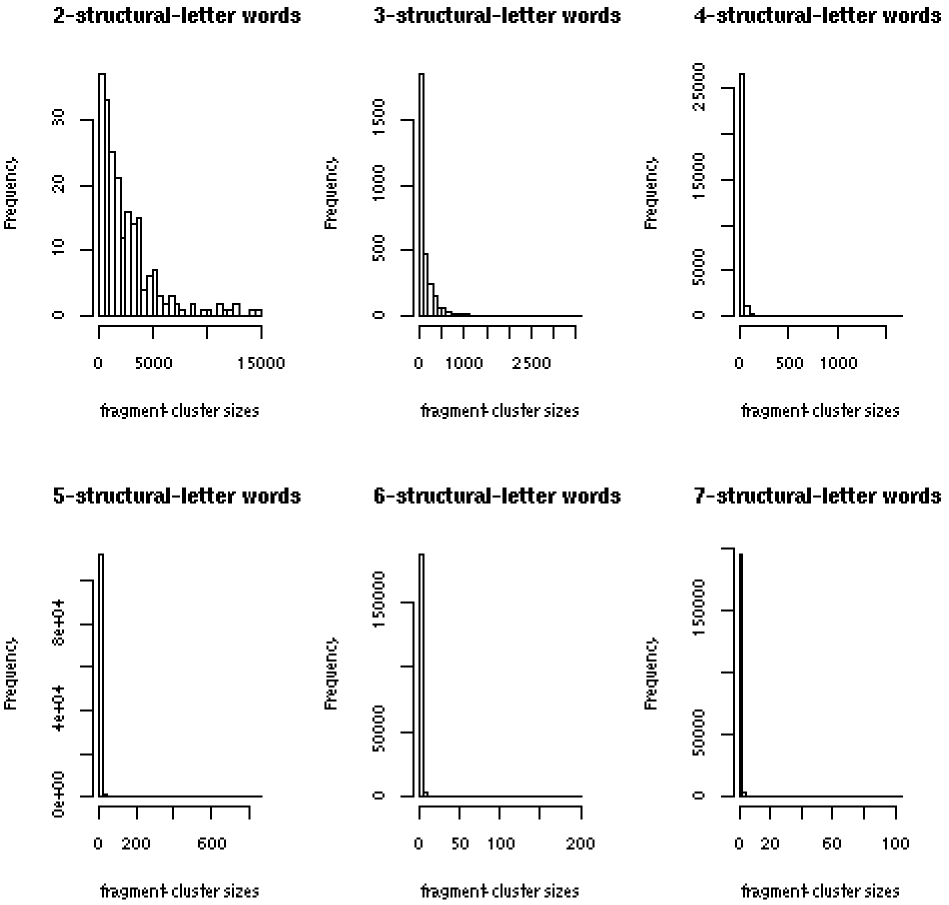

Figure 1: Distribution of occurrences of loop words with length from 2-structural letters (5 residues) from 7-structural letters (10 residues)

2- Comparison of the loop-length distribution in loop set containing all words and loop set containing only words seen 30 times

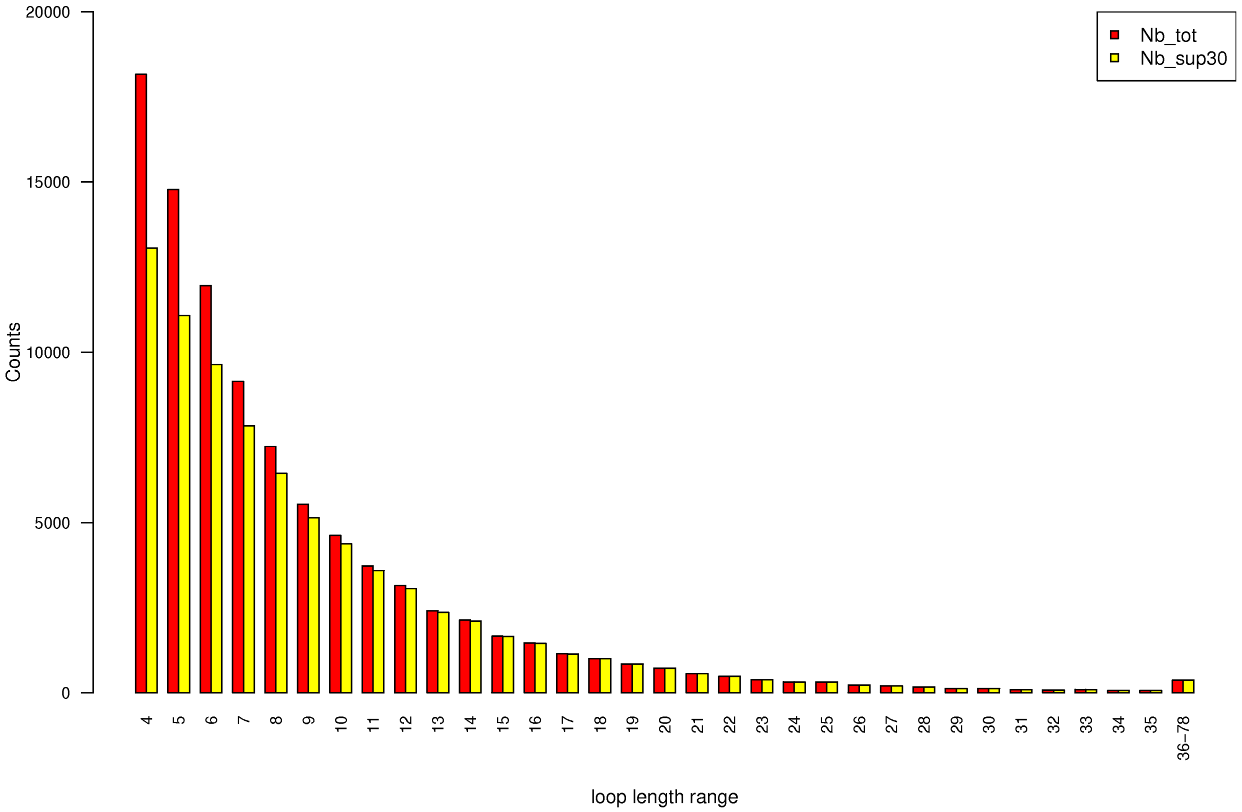

Figure 2: Length distribution of loops from which structural words are extracted. Red bars: length distribution of all loops in the dataset. Yellow bars: length distribution of loops from which $Wset_{\geq 30}$ *are extracted. Lengths are expressed in terms of structural letters.*

3- Robustness of the word statistical analysis on different data sets

Comparison of the words statistics computed in a bank presenting less than:
• 25% and 50% of sequence identity.
• 80% and 50% of sequence identity.

3.1- Dataset description

In order to compare the robustness of the word statistics across different datasets, we build three datasets of simplified loops. Loops are extracted from
• dataset of proteins presented less than 25% of sequence identity (Dataset25, 05/2007)
• dataset of proteins presented less than 50% of sequence identity (Dataset50, 06/2008)
• dataset of proteins presented less than 80% of sequence identity (Dataset80, 06/2006)

We compare the statistics of the 22560 common words in the 3 datasets by computing the consensus level which corresponds to the proportion of structural words – common to both datasets – that are classified in the same statistical word type (over-represented/not significant/under-represented).

3.2- Comparison of the 22560 word statistics

3.2.1- Dataset25 vs Dataset50

The comparison of word statistics in the Dataset25 and Dataset50 results in a consensus level of 79% :
• 95 % of not significant words in Dataset50 are not significant in Dataset25
• 66 % of under-represented words in Dataset50 are under-represented in Dataset25
• 75 % of over-represented words in Dataset50 are over represented in Dataset25
Table 1 presents the contingency table of this comparison.

	Dataset50				sum
		NS	UR	OR	20191
Dataset25	NS	19677	234	280	895
	UR	435	459	1	1474
	OR	615	0	859	
sum		20727	693	1140	22560

Table 2: Comparison of the word number in each word statistic types in datasets with less than 25 % (Dataset25) and 50 % (Dataset50) of sequence identity. NS= not significant words; UR= under-represented words; OR = over-represented words.

3.2.2- Dataset80 vs Dataset50

The comparison of word statistics in the Dataset85 and Dataset50 results in a consensus level of 90% :
• 98 % of not significant words in Dataset50 are not significant in Dataset80

• 83 % of under-represented words in Dataset50 are under-represented in Dataset80
• 89 % of over-represented words in Dataset50 are over represented in Dataset80
Table 2 presents the contingency table of this comparison.

	Dataset50				sum
Dataset80		NS	UR	OR	20552
	NS	20285	78	189	762
	UR	147	615	0	1246
	OR	295	0	951	
sum		20727	693	1140	22560

Table 3: Comparison of the word number in each word statistic types in datasets with less than 80 % (Dataset80) and 50 % (Dataset50) of sequence identity. NS= not significant words; UR= under-represented words; OR = over-represented words.

3.3- Comparison of the 890 words seen 30 times in Dataset50

3.3.1- Dataset25 vs Dataset50

The comparison of word statistics in the Dataset25 and Dataset50 results in a consensus level of 82% :
• 85 % of not significant words in Dataset50 are not significant in Dataset25
• 88 % of under-represented words in Dataset50 are under-represented in Dataset25
• 76 % of over-represented words in Dataset50 are over represented in Dataset25
Table 3 presents the contingency table of this comparison.

	Dataset50				sum
Dataset25		NS	UR	OR	2189
	NS	1856	27	228	196
	UR	155	145	1	925
	OR	175	0	723	
sum		2111	301	898	3310

Table 4: Comparison of the word number in each word statistic types in datasets with less than 25 % (Dataset25) and 50 % (Dataset50) of sequence identity. The statistics were computed on words seen more 30 times in Dataset50 and seen in Dataset25. NS= not significant words; UR= under-represented words; OR = over-represented words.

3.3.2- Dataset80 vs Dataset50

The comparison of word statistics in the Dataset80 and Dataset50 results in a consensus level of 90% :
• 93 % of not significant words in Dataset50 are not significant in Dataset80
• 97 % of under-represented words in Dataset50 are under-represented in Dataset80
• 85 % of over-represented words in Dataset50 are over represented in Dataset80
Table 4 presents the contingency table of this comparison.

<table>
<tr><td></td><td colspan="4">Dataset50</td><td>sum</td></tr>
<tr><td rowspan="4">Dataset80</td><td></td><td>NS</td><td>UR</td><td>OR</td><td>2189</td></tr>
<tr><td>NS</td><td>2036</td><td>13</td><td>140</td><td>196</td></tr>
<tr><td>UR</td><td>37</td><td>159</td><td>0</td><td rowspan="2">925</td></tr>
<tr><td>OR</td><td>113</td><td>0</td><td>812</td></tr>
<tr><td colspan="2">sum</td><td>2186</td><td>172</td><td>952</td><td>3310</td></tr>
</table>

Table 5: Comparison of the word number in each word statistic types in datasets with less than 80 % (Dataset80) and 50 % (Dataset50) of sequence identity. The statistics were computed on words seen more 30 times in Dataset50 and seen in Dataset25. NS= not significant words; UR= under-represented words; OR = over-represented words.

4- Coverage of SCOP superfamilies by recurrent words

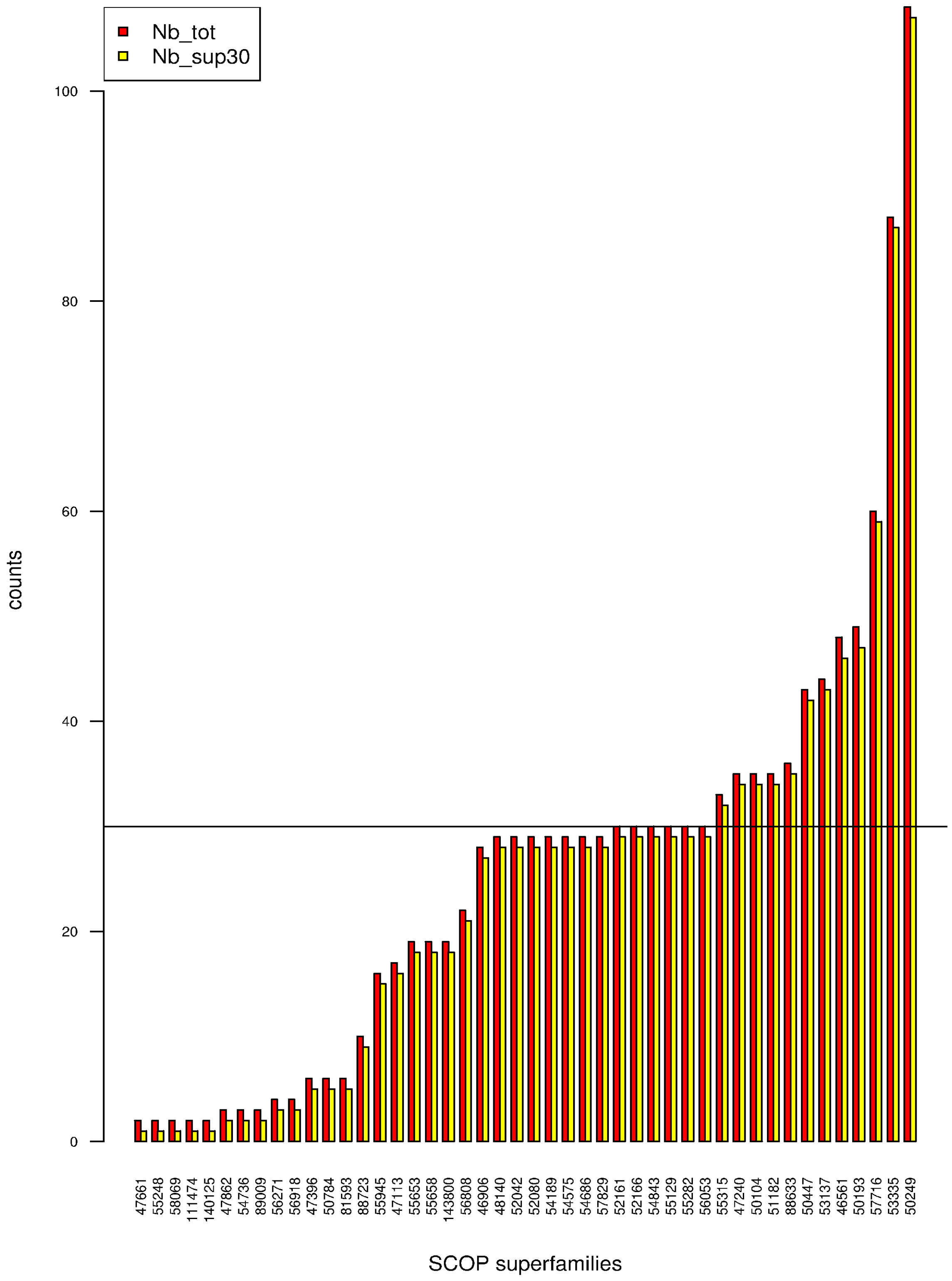

Figure 3: Distribution of the protein superfamilies affected by the removing of rare words. Red bars: Superfamily distribution of all loops in the dataset. Yellow bars: Superfamily distribution of loops from which $Wset_{\geq 30}$ are extracted. Protein superfamilies are extracted from the protein classification SCOP. The horizontal line corresponds to a threshold of 30 occurrences.

5- Correlation between sequence specificity (Z_{max}) and structure variability ($RMSd_w$) for all words in $Wset_{\geq 30}$

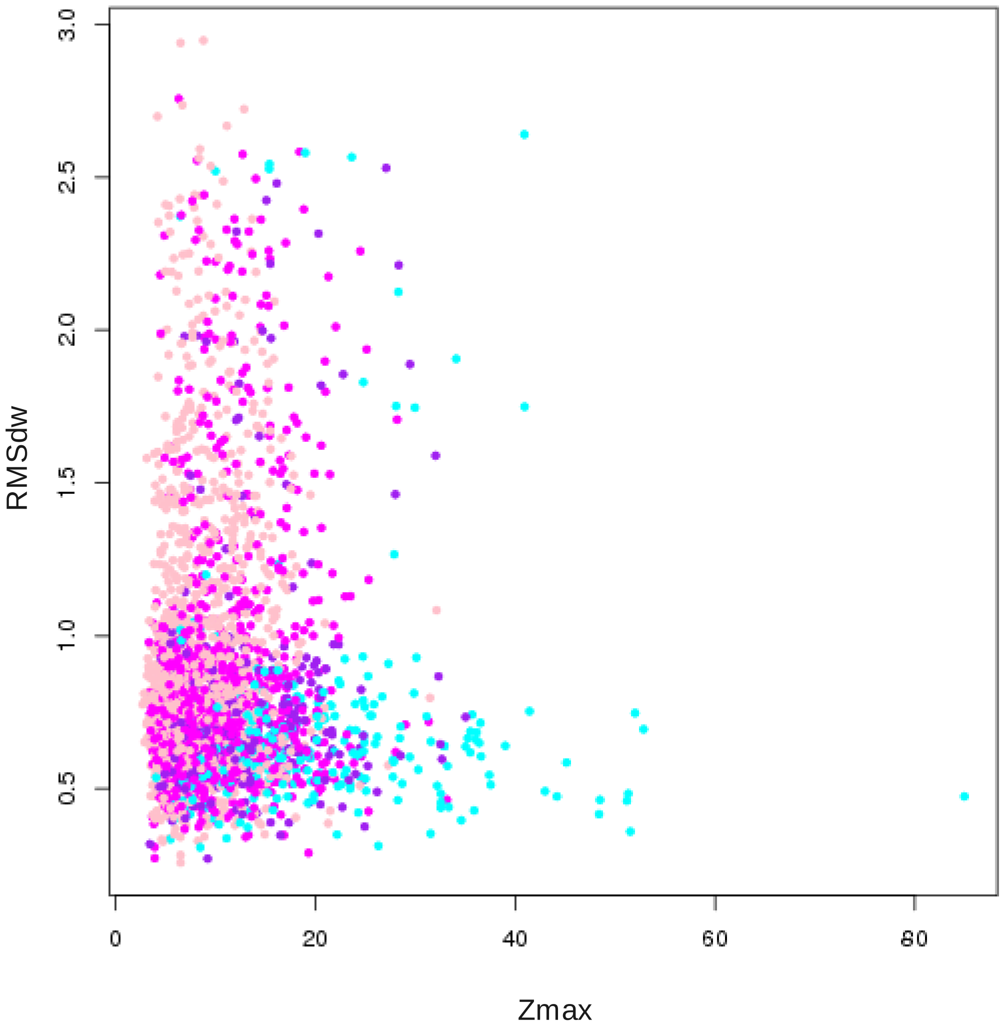

Figure 4: Plot of sequence specificity (Zmax) versus structure variability (RMSdw) for all words in $Wset_{\geq 30}$. Words are colored according to their occurrence (occ): pink: occ=30 to 50, magenta: occ=50 to 100, purple: occ=100 to 150, cyan: occ ≥150

6- Exceptionality score L_p *versus* frequency for the 28274 words of the dataset

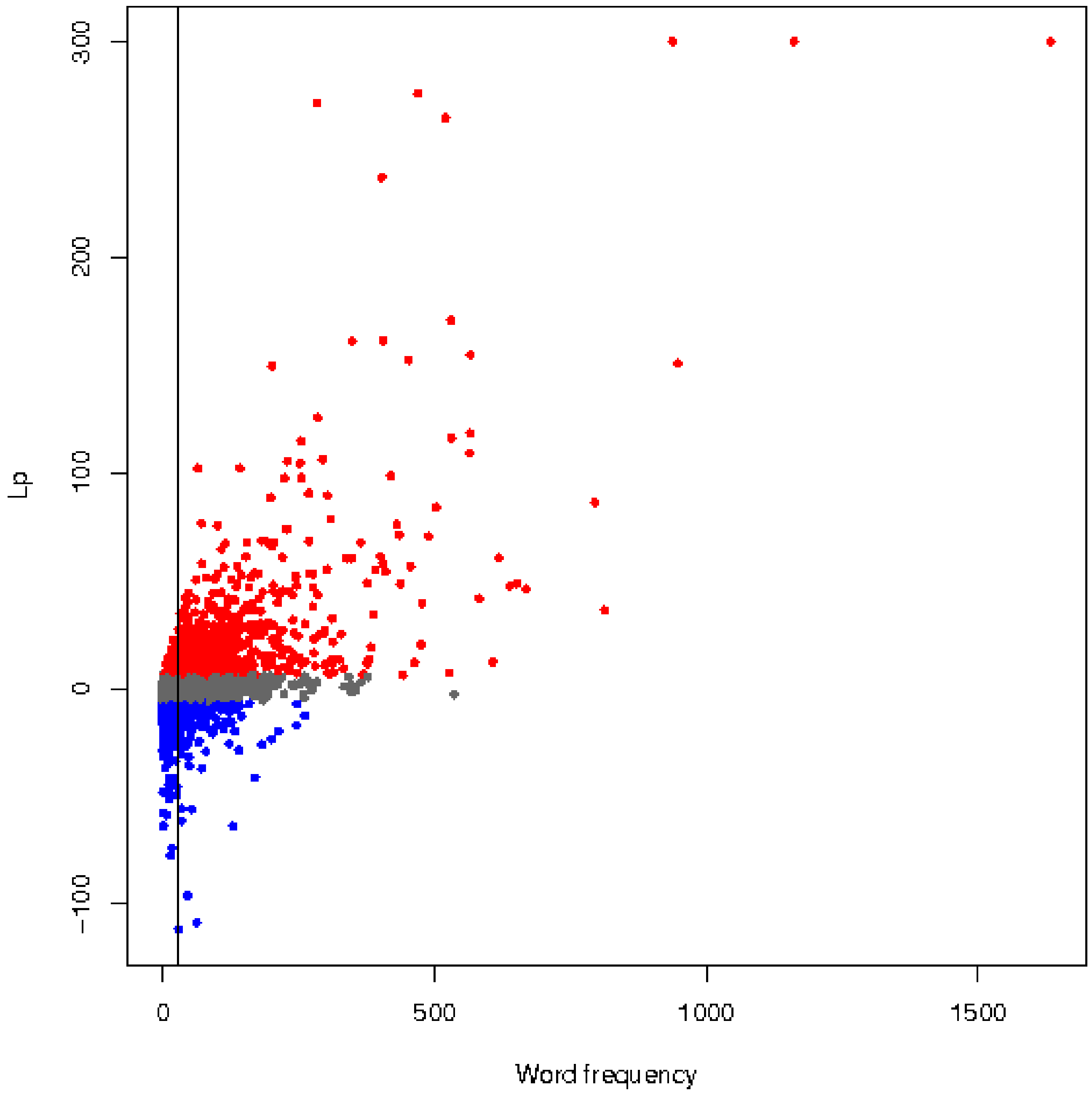

Figure 5: Exceptionality score Lp versus frequency for the 28274 words of the data set. Words are colored according to their statistical type: red=over-represented words, gray=not significant words and blue=under-represented words. The vertical line corresponds to the frequency threshold of 30.

7- ClustalW of 3SIL sequence (**P29768)** + homologuous sequences from UniProt

```
                    10        20        30        40        50        60
                     |         |         |         |         |         |
P10481       ------------------------------------------------------------
P15698       ------------------------------------------------------------
P29768       ------------------------------------------------------------
P31206       ------------------------------------------------------------
P23253       MVAIADARYETSSENSLIDTVAKYSVDDGETWETQIAIKNSRVSSVSRVVDPTVIVKGNK
Q27701       ------------------------------------------------------------

Prim.cons.   MVAIADARYETSSENSLIDTVAKYSVDDGETWETQIAIKNSRVSSVSRVVDPTVIVKGNK

                    70        80        90       100       110       120
                     |         |         |         |         |         |
P10481       ------------------------------------------------------------
P15698       ------------------------------------------------------------
P29768       ------------------------------------------------------------
P31206       ------------------------------------------------------------
P23253       LYVLVGSYYSSRSYWSSHGDARDWDILLAVGEVTKSTAGGKITASIKWGSPVSLKKFFPA
Q27701       ------------------------------------------------------------

Prim.cons.   LYVLVGSYYSSRSYWSSHGDARDWDILLAVGEVTKSTAGGKITASIKWGSPVSLKKFFPA

                   130       140       150       160       170       180
                     |         |         |         |         |         |
P10481       ------------------------------------------------------------
P15698       ------------------------------------------------------------
P29768       ------------------------------------------------------------
P31206       ------------------------------------------------------------
P23253       EMEGMHTNQFLGGAGVAIVASNGNLVYPVQVTNKRKQVFSKIFYSEDDGKTWKFGKGRSD
Q27701       ------------------------------------------------------------

Prim.cons.   EMEGMHTNQFLGGAGVAIVASNGNLVYPVQVTNKRKQVFSKIFYSEDDGKTWKFGKGRSD

                   190       200       210       220       230       240
                     |         |         |         |         |         |
P10481       ------------------------------------------------------------
P15698       ------------------------------------------------------------
P29768       ------------------------------------------------------------
P31206       ------------------------------------------------------------
P23253       FGCSEPVALEWEGKLIINTRVDWKRRLVYESSDMEKPWVEAVGTVSRVWGPSPKSNQPGS
Q27701       ------------------------------------------------------------

Prim.cons.   FGCSEPVALEWEGKLIINTRVDWKRRLVYESSDMEKPWVEAVGTVSRVWGPSPKSNQPGS

                   250       260       270       280       290       300
                     |         |         |         |         |         |
P10481       ------------------------------------------------------------
P15698       ------------------------------------------------------------
P29768       ------------------------------------------------------------
P31206       ------------------------------------------------------------
P23253       QTSFTAVTIEGMRVMLFTHPLNFKGRCVRDRLNLWLTDNQRIYNVGQVSIGDENSAYSSV
Q27701       ------------------------------------------------------------

Prim.cons.   QTSFTAVTIEGMRVMLFTHPLNFKGRCVRDRLNLWLTDNQRIYNVGQVSIGDENSAYSSV
```

```
                       310       320       330       340       350       360
                        |         |         |         |         |         |
P10481         ------------------------------------------------------------
P15698         ------------------------------------------------------------
P29768         ------------------------------------------------------------
P31206         ------------------------------MKKAVILFSLFCFLCAIPVVQAADTIFVRE
P23253         LYKDDKLYCLHEINTDEVYSLVFARLVGELRIIKSVLRSWKNWTATCPAFAPLLIQPLRR
Q27701         -------------MGRIGKKAMAIALVSAVMVTPLNVCATVENQEQQQVTQGAEDIAVID

Prim.cons.     LYKDDKLYCLHEI222222222222LV222M33333L3S33333333PV3Q3A33I3VR3

                       370       380       390       400       410       420
                        |         |         |         |         |         |
P10481         ------------------------------------------------------------
P15698         -----------------------------------------------------------M
P29768         ------------------------------------------------------------
P31206         TRIPILIERQDNVLFYLRLDAKESQTLNDVVLNLGEGVNLSEIQSIKLYYGGTEALQDSG
P23253         QRVVVVPLSPRLVLLAFCRQRLPKRMGGSYRCVNASTANAERVRNGLKFAGVGGGALWPV
Q27701         DAQETVAADEAQADEAAAITVEGRETAEESSASIPEGILMEKNNVDIAEGQGYSLDQEAG

Prim.cons.     3R333V333333VL3A333333333T333333333EG3N3E333333333GG3333Q33G

                       430       440       450       460       470       480
                        |         |         |         |         |         |
P10481         -----------------MCNKNNTFEKN--------------------------------
P15698         KKFIKILKVLSMAIVLSACNINGIFASN--------------------------------
P29768         ----------------MTVEKSVVFKAE--------------------------------
P31206         KKRFAPVGYISSNTPGKTLAANPSYSIK--------------------------------
P23253         SQQGQNQRYRFANHAFTLVASVTIHEAPRA------------------------------
Q27701         AKYVKAMTQGTIILSYKSTSENGIQSLFSVGNSTAGNQDRHFHIYITNSGGIGIELRNTD

Prim.cons.     KK44K444Y4S4N444KT22KNGIF2AN22GNSTAGNQDRHFHIYITNSGGIGIELRNTD

                       490       500       510       520       530       540
                        |         |         |         |         |         |
P10481         ----------------------------LDISHKPEPLILFNK-----------------
P15698         ----------------------------LNTTNEPQKTTVFNK-----------------
P29768         ----------------------------GEHFTDQKGNTIVG------------------
P31206         ----------------------------KSEVTNPGNQVVLKGDQKLFPGINYFWISLQM
P23253         -----------------------ASPLLGASLDSSGGKKLLGLSYDEKHQWQPIYGSTPV
Q27701         GVFNYTLDRPASVRALYKGERVFNTVALKADAANKQCRLFANGELLATLDKDAFKFISDI
                                                .     .
Prim.cons.     GVFNYTLDRPASVRALYKGERVF2222L3A66TNP2G6T22N2333333333F33S333

                       550       560       570       580       590       600
                        |         |         |         |         |         |
P10481         -------------------------------------------------------DNNIW
P15698         -------------------------------------------------------NDNTW
P29768         -------------------------------------------------------SGSGG
P31206         KPGTSLTSKVTADIASITLDGKKAL------------LDVVSENGIEHRMGVGVRHAGDD
P23253         TPTGSWETG-KRYHLVLTMANKIGS------------VYIDGELLEGSGQTVVPDGRTPD
Q27701         TGVDNVTLGGTKRQGKIAYPFGGTIGDIKVYSNALSDEELIQATGVTTYGENIFYAGDVT

Prim.cons.     TP33S3T3G2T33333IT333K333GDIKVYSNALSD33333E3G333333V3336GN62

                       610       620       630       640       650       660
                        |         |         |         |         |         |
P10481         NSKYFR-------IPNIQLLNDGTILTFSDIRYNGPDD------------HAYIDIASAR
P15698         NAQYFR-------IPSLQTLADGTMLAFSDIRYNGAED------------HAYIDIGAAK
P29768         TTKYFR-------IPAMCTTSKGTIVVFADARHNTASD------------QSFIDTAAAR
P31206         NSAAFR-------IPGLVTTNKGTLLGVYDVRYNSSVDL-----------QEHVDVGLSR
```

```
P23253       ISHFYVGGYKRSDMPTISHVTVNNVLLYNRRQLNTEEIRTLFLSQDLIGTEAHMDSSSDS
Q27701       ESNYFR-------IPSLLTLSTGTVISAADARYGGTHDS-----------KSKINIAFAK
              :  :        :* :     ..::     : .                .  :: .
Prim.cons.   NSKYFRGGYKRSDIPSLQTL22GT2L6F2D2RYNGAED3TLFLSQDLIGT2A2IDIA2AR

                    670       680       690       700       710       720
                      |         |         |         |         |         |
P10481       STDFGKTWSYN-------------IAMKNNRIDS-----TYSRVMDSTTVITNTG-----
P15698       STDNGQTWDYK-------------TVMENDRIDS-----TFSRVMDSTTVVTDTG-----
P29768       STDGGKTWNKK-------------IAIYNDRVNS-----KLSRVMDPTCIVANIQG----
P31206       STDGGKTWEKMR---------LPLAFGEFGGLPA-----GQNGVGDPSILVDTKT-----
P23253       SAHSTPSTPADSSAHSTPSTPVDSSAHSTPSTPA-----DSSAHGTPSTPVDSSAHGTPS
Q27701       STDGGNTWSEPTLPLKFDDYIAKNIDWPRDSVGKNVQIQGSASYIDPVLLEDKLTKRIFL
             *:.   :                                       .
Prim.cons.   STDGGKTWS2K3222222222333IAMENDR22SNVQIQ2SSRVMDPTT2VDNT232222

                    730       740       750       760       770       780
                      |         |         |         |         |         |
P10481       ------------------------RIILIAGSWNTNGN--WAMTTSTRRSDWS-------
P15698       ------------------------RIILIAGSWNKNGN--WASSTTSLRSDWS-------
P29768       ----------------------RETILVMVGKWNNNDKTWGAYRDKAPDTDWD-------
P31206       ------------------------NNVWVVAAWTHGMGNQRAWWSSHPGMDMNHTA----
P23253       TPADSSA-------------HGTPSTPVDSSAHSTPSTPVDSSAHSTPSTPVDSSA----
Q27701       FADLMPAGIGSSNASVGSGFKEVNGKKYLKLRWHKDAGRAYDYTIREKGVIYNDATNQPT
                                                                 .
Prim.cons.   222222AGIGSSNASVGSGF2233RII2I2G2WN2NG244WA2TTSTP22DW333ANQPT

                    790       800       810       820       830       840
                      |         |         |         |         |         |
P10481       ----------------------------------------------------------VQ
P15698       ----------------------------------------------------------VQ
P29768       ----------------------------------------------------------LV
P31206       ---------------------------------------------------------QLV
P23253       ---------------------------------------------------------HGA
Q27701       EFRVDGEYNLYQHDTNLTCKQYDYNFSGNNLIESKTDVDVNMNIFYKNSVFKAFPTNYLA

Prim.cons.   EFRVDGEYNLYQHDTNLTCKQYDYNFSGNNLIESKTDVDVNMNIFYKNSVFKAFPTN3L3

                    850       860       870       880       890       900
                      |         |         |         |         |         |
P10481       MIYSDDNGLTWSNKIDLTKDSSKVKNQPSNTIGWLGGVGSGIVMDDGTIVMPAQISLREN
P15698       MVYSDDNGETWSDKVDLTTNKARIKNQPSNTIGWLAGVGSGIVMSDGTIVMPIQIALREN
P29768       LYKSTDDGVTFS---KVETNIHDIVTKNGTISAMLGGVGSGLQLNDGKLVFPVQMVRTKN
P31206       LAKSTDDGKTWS----APINITEQVKDPS-WYFLLQGPGRGITMSDGTLVFPTQFIDSTR
P23253       PSTPADSSAHGTPSTPVDSSAHGTPSTPADSSAHSTPSTPADSSAHSTPSTPADSSAHST
Q27701       MRYSDDEGASWS----DLDIVSSFKPEVSKFLVVGPGIGKQISTGENAGRLLVPLYSKSS
                . *..   :                .                ..
Prim.cons.   M6YSDD2GATWS3K3D2TTNI26IKNQPSNT22WLGGVGSGI2MSDGT2V2P2QISLR2N

                    910       920       930       940       950       960
                      |         |         |         |         |         |
P10481       NENNYYSLIIYSKDN---GETWTMGNK--VPNSNTSENMVIELD--GALIMSTRYDYSGY
P15698       NANNYYSSVIYSKDN---GETWTMGNK--VPDPKTSENMVIELD--GALIMSSRNDGKNY
P29768       ITTVLNTSFIYSTD----GITWSLPSG--YCEGFGSENNIIEFN--ASLVNNIRN--SGL
P31206       VPN---AGIMYSKDG---GKNWKMHN---YARTNTTEAQVAEVEP-GVLMLNMRDNRGGS
P23253       PSTPVDSSAHGTPSTPADSSAHSTPST--PADSSAHGTPSTPVDSSAHSTPSTPVDSSAH
Q27701       AELG----FMYSDDH---GDNWTYVEADNLTGGATAEAQIVEMPD-GSLKTYLRTG-SNC
                        : .    .   .  .                .   .
Prim.cons.   NENNYYSS2IYSKDNPADGETWTM2NKDN22D2NTSEN2VIE2D3SG2LIMSTRND4SGY

                    970       980       990      1000      1010      1020
```

```
                      |         |         |         |         |         |
P10481       RAAYISHDLGTTWEIYEPLNGKILTGKGSGCQGSFIKATTSNG-HRIGLISAPKNTKGEY
P15698       RASYISYDMGSTWEVYDPLHNKISTGNGSGCQGSFIKVTAKDG-HRLGFISAPKNTKGGY
P29768       RRSFETKDFGKTWTEFPPMDKKVDNRN-HGVQGSTITIPSGN--KLVAAHSSAQNKNNDY
P31206       RAVAITKDLGKTWTEHESSRKALPESVCMASLISVKAKDNVLG-KDLLIFSNPNTTKGRY
P23253       GTPSTPVDSSAHSTPSTPVDSSAHGTPSTPVDSSAHSTPSTP--ADSSAHSTPSTPADSS
Q27701       IAEVTSIDGGETWSDRVPLQGISTTSY--GTQLSVINYSQPIDGKPAIILSSPNATNGRK
                  . * .       .               *                * ..   .
Prim.cons.   RASYISKDLGKTWTEYEPLD2KI6T2NGSG2QGS2IK62S6NGGK2LG22S2P2NTKGRY

                   1030      1040      1050      1060      1070      1080
                      |         |         |         |         |         |
P10481       IR----DNIAVYMIDFDDLS---KGVQEICIPYPEDGNKLGGGYSCLSFKN----NHLGI
P15698       VR----DNITVYMIDFDDLS---KGIRELCSPYPEDGNSSGGGYSCLSFND----GKLSI
P29768       TR----SDISLYAHNLYSG-----EVKLIDDFYPKVGNASGAGYSCLSYRKNVDKETLYV
P31206       N--------TTIKISLDGG----VTWSPEHQLLLDEGNNWG--YSCLSMID---KETIGI
P23253       AHGTPSTPVDSSAHSTPSTP---ADSSAHSTPSTPVDSSAHSTPSTPADSSAHGTPSTPV
Q27701       NG-----KIWIGLVNDTGNTGIDKYSVEWKYSYAVDTPQMGYSYSCLAELP---DGQVGL
                           .  .                          *  :           :
Prim.cons.   NRGTPSDNITVY2I32D32SGIDKG2SEIC6PYPEDGNSSGGGYSCLSF6D222K2TLGI

                   1090      1100      1110      1120      1130      1140
                      |         |         |         |         |         |
P10481       VYEANGNIEYQDLTPYYSLINKQ-------------------------------------
P15698       LYEANGNIEYKDLTDYYLSIENNKKLK---------------------------------
P29768       VYEANGSIEFQDLSRHLPVIKSYN------------------------------------
P31206       LYESSVAHMTFQAVKLKDIIK---------------------------------------
P23253       DSSAHGTPSTPADSSAHSTPSTPADSSAHSTPSTPADSSAHSTPSTPVDSSAHSTPSTPA
Q27701       LYEKYDSWSRNELHLKDILKFEKYSISELTGQASGN------------------------
               .
Prim.cons.   LYEANG2IE2QDL26YYSLIK55433S222222222DSSAHSTPSTPVDSSAHSTPSTPA

                   1150      1160      1170      1180      1190      1200
                      |         |         |         |         |         |
P10481       ------------------------------------------------------------
P15698       ------------------------------------------------------------
P29768       ------------------------------------------------------------
P31206       ------------------------------------------------------------
P23253       DSSAHSTPSTPADSSAHSTPSTPADSSAHSTPSTPVDSSAHSTPSTPADSSAHGTPSTPA
Q27701       ------------------------------------------------------------

Prim.cons.   DSSAHSTPSTPADSSAHSTPSTPADSSAHSTPSTPVDSSAHSTPSTPADSSAHGTPSTPA

                   1210      1220      1230      1240      1250      1260
                      |         |         |         |         |         |
P10481       ------------------------------------------------------------
P15698       ------------------------------------------------------------
P29768       ------------------------------------------------------------
P31206       ------------------------------------------------------------
P23253       DSSAHSTPSTPVDSSAHSTPSTPADSSAHGTPSTPADSSAHSTPSTPADSSAHGTPSTPA
Q27701       ------------------------------------------------------------

Prim.cons.   DSSAHSTPSTPVDSSAHSTPSTPADSSAHGTPSTPADSSAHSTPSTPADSSAHGTPSTPA

                        1270      1280      1290      1300      1310
                           |         |         |         |         |
   P10481       ------------------------------------------------------
   P15698       ------------------------------------------------------
   P29768       ------------------------------------------------------
   P31206       ------------------------------------------------------
   P23253       DSSAHSTPSTPAGSSANGTVLILPDGAALSTFSGGGLLLCACALLLHVFFMAVF
```

```
    Q27701       -------------------------------------------------------

Prim.cons.   DSSAHSTPSTPAGSSANGTVLILPDGAALSTFSGGGLLLCACALLLHVFFMAVF
```

ClustalW of 3SIL sequence + homologuous sequences from PDB

```
                       10        20        30        40        50        60
                        |         |         |         |         |         |
3SILxx0        ------------------------------------------------------------
pdb1dilA       ------------------------------------------------------------
pdb1n1tA       ------------------------------------------------------------
pdb2bf6A       ------------------------------------------------------------
pdb2sliA       IPEGILMEKNNVDIAEGQGYSLDQEAGAKYVKAMTQGTIILSYKSTSENGIQSLFSVGNS
pdb3b69A       ------------------------------------------------------------
pdb3h73B       ------------------------------------------------------------

Prim.cons.     IPEGILMEKNNVDIAEGQGYSLDQEAGAKYVKAMTQGTIILSYKSTSENGIQSLFSVGNS

                       70        80        90       100       110       120
                        |         |         |         |         |         |
3SILxx0        ------------------------------------------------------------
pdb1dilA       ------------------------------------------------------------
pdb1n1tA       ------------------------------------------------------------
pdb2bf6A       ------------------------------------------------------------
pdb2sliA       TAGNQDRHFHIYITNSGGIGIELRNTDGVFNYTLDRPASVRALYKGERVFNTVALKADAA
pdb3b69A       ------------------------------------------------------------
pdb3h73B       ------------------------------------------------------------

Prim.cons.     TAGNQDRHFHIYITNSGGIGIELRNTDGVFNYTLDRPASVRALYKGERVFNTVALKADAA

                      130       140       150       160       170       180
                        |         |         |         |         |         |
3SILxx0        ------------------------------------------------------------
pdb1dilA       -----------------------------------------------------------T
pdb1n1tA       ------------------------------------------------------AASLAPG
pdb2bf6A       ------------------------------------------------------------
pdb2sliA       NKQCRLFANGELLATLDKDAFKFISDITGVDNVTLGGTKRQGKIAYPFGGTIGDIKVYSN
pdb3b69A       -------------------------------------------------------ASLAPG
pdb3h73B       ------------------------------------------------------------

Prim.cons.     NKQCRLFANGELLATLDKDAFKFISDITGVDNVTLGGTKRQGKIAYPFGGTIG2ASLAPG

                      190       200       210       220       230       240
                        |         |         |         |         |         |
3SILxx0        --KSVVFKAEGEHFTDQKGNTIVGSGSGGTTK---YFRIPAMCTTSKGTIVVFADARHNT
pdb1dilA       VEKSVVFKAEGEHFTDQKGNTIVGSGSGGTTK---YFRIPAMCTTSKGTIVVFADARHNT
pdb1n1tA       SSRVELFKRKNSTVPFEES---NGTIRERVVH---SFRIPTIVN-VDGVMVAIADARYET
pdb2bf6A       --------VEGAVKTEPVDLFHPGFLNSSN------YRIPALFKTKEGTLIASIDARRHG
pdb2sliA       ALSDEELIQATGVTTYGENIFYAGDVTESN-----YFRIPSLLTLSTGTVISAADARYGG
pdb3b69A       SSRVELFKRQSSKVPFEK----DGKVTERVVH---SFRLPALVN-VDGVMVAIADARYET
pdb3h73B       -------LPEGAALTEKTDIFESGRNGKPNKDGIKSYRIPALLKTDKGTLIAGADERRLH
                             .        *            :*:*:: .   *.::   * *
Prim.cons.     SS22E2FK2EG322T32K22FIVGS22E3N22GIK2FRIPAL3TTSKGT3VA2ADARY2T

                      250       260       270       280       290       300
                        |         |         |         |         |         |
3SILxx0        ASDQS--FIDTAAARSTDGGKTWNKKIAIYNDRVNSKLSRVMDPTCIVAN----IQGRET
pdb1dilA       ASDQS--FIDTAAARSTDGGKTWNKKIAIYNDRVNSKLSRVMDPTCIVAN----IQGRET
pdb1n1tA       SFDNS--FIETAVKYSVDDGATWNTQIAIKNSRASS-VSRVMDATVIVK------GNK--
pdb2bf6A       GADAPNNDIDTAVRRSEDGGKTWDEGQIIMDYPD---KS---------------S--V-
pdb2sliA       THDSKS-KINIAFAKSTDGGNTWSEPTLPLKFDDYI-AKNIDWPRDSVGKNVQIQGSASY
pdb3b69A       SFDNS--LIDTVAKYSVDDGETWETQIAIKNSRASS-VSRVVDPTVIVK------GNK--
pdb3h73B       SSDWG--DIGMVIRRSEDNGKTWGDRVTITNLRDNP-KASDPS-----------IGSPVN
                 *     *  .   * *.* **       .
```

```
Prim.cons.   SSD2S2NFIDTAAARSTDGGKTWN32IAI2N2RDNSK3SRVMDPT2IV2NNVQIIG322T

                    310       320       330       340       350       360
                      |         |         |         |         |         |
3SILxx0      ILVMVGKWNNNDKTWGAYRDKAPDTDWDLVLYK-----STDDGVTFSKVETN-------I
pdb1dilA     ILVMVGKWNNNDKTWGAYRDKAPDTDWDLVLYK-----STDDGVTFSKVETN-------I
pdb1n1tA     LYILVGSFNKTRNSWTQHRD-GS--DWEPLLVVGEVTKSAANGKTTATISWGKPVSLKPL
pdb2bf6A     IDTTLIQDDETGRIFLLVTHFPSKYGFWNAG--LGSGFKNIDGKEYLCLYDSSGK----E
pdb2sliA     IDPVLLEDKLTKRIFLFADLMPAGIGSSNAS--VGSGFKEVNGKKYLKLRWHKDAGRAYD
pdb3b69A     LYVLVGSYNSSRSYWTSHGD-AR--DWDILLAVGEVTKSTAGGKITASIKWGSPVSLKEF
pdb3h73B     IDMVLVQDPETKRIFSIYDMFPEGKGIFGMSSQKEEAYKKIDGKTYQILYREGEKG---A
             :   : .   .   :          .            .  .*     :
Prim.cons.   IDV3VG3DN2T3RIW3AYRD2222TDWD23LY2GE222ST3DGKTY3KL2W22P22LK3I

                    370       380       390       400       410       420
                      |         |         |         |         |         |
3SILxx0      HDIVTKNGTISAMLGGVGSGLQLNDGKLVFPVQMVRTKNITTVLNTSFIYSTDGITWSLP
pdb1dilA     HDIVTKNGTISAMLGGVGSGLQLNDGKLVFPVQMVRTKNITTVLNTSFIYSTDGITWSLP
pdb1n1tA     FPAEFDGILTKEFVGGVGAAIVASNGNLVYPVQIADMG--GRVFTKIMYSEDDGNTWKFA
pdb2bf6A     FTVREN-VVYDKDSNKTEY-----------T-----TNALGDLFKNGTKIDNINSST---
pdb2sliA     YTIREKGVIYNDATNQPTEFRVDGEYNLYQHDTNLTCKQYDYNFSGNNLIESKTDVDVNM
pdb3b69A     FPAEMEGMHTNQFLGGAGVAIVASNGNLVYPVQVTNKK--KQVFSKIFYSEDEGKTWKFG
pdb3h73B     YTIRENGTVYTPDGKATDYRVVV-------DPVKPAYSDKGDLYKGNQLLGNIYFTTN--
             .    .                                      .
Prim.cons.   FTIREKG22Y2A3LGGVG222V222GNLV2PVQMVRTKNIG2VF333F33E3DGITW22P

                    430       440       450       460       470       480
                      |         |         |         |         |         |
3SILxx0      SGYCEGFGSENNIIEFNASLVNNIRNSGLRR-SFETKDFGKTWTEFP-----PMDKKVDN
pdb1dilA     SGYCEGFGSENNIIEFNASLVNNIRNSGLRR-SFETKDFGKTWTEFP-----PMDKKVDN
pdb1n1tA     EGRSKFGCSEPAVLEWEGKLIINNRVDGNRRLVYESSDMGKTWVEALGTLSHVWTNSPTS
pdb2bf6A     ------APLKAKGTSYINLVYSDDDGKTWSEPQNINFQVKKDWMKFLGIAPGRGIQIKNG
pdb2sliA     NIFYKNSVFKAFPTNYLAMRYSDDEGASWS-DLDIVSSFKPEVSKFLVVGPGIGKQISTG
pdb3b69A     KGRSAFGCSEPVALEWEGKLIINTRVDYRRRLVYESSDMGNTWLEAVGTLSRVWGPSPKS
pdb3h73B     ----KTSPFRIAKDSYLWMSYSDDDGKTWSAPQDITPMVKADWMKFLGVGPGTGIVLRNG
                      .    .:      :               .     :              .
Prim.cons.   SG22K233SE32I3EY3A3LYSNDRG3GWRR233ETSDFGKTW2EFLG22PG2G22323G

                    490       500       510       520       530       540
                      |         |         |         |         |         |
3SILxx0      RNHGVQGSTITIPS-----GNKLVAAHSSAQNKNNDYTRSDISLYAHN------------
pdb1dilA     RNHGVQGSTITIPS-----GNKLVAAHSSAQNKNNDYTRSDISLYAHN------------
pdb1n1tA     NQQDCQSSFVAVTI-----EGKRVMLFTHPLNLKGRWMRDRLHLWMTDNQRIFDVGQISI
pdb2bf6A     EHKGRIVVPVYYTN----EKGKQSSAVIYSDDSGKNWTIGESPNDNRKLEN---------
pdb2sliA     ENAGRLLVPLYSKS-------SAELGFMYSDDHGDNWTYVEADNLTGGAT----------
pdb3b69A     NQPGSQSSFTAVTI-----EGMRVMLFTHPLNFKGRWLRDRLNLWLTDNQRIYNVGQVSI
pdb3h73B     PHKGRILIPVYYTTNNVSHLNGSQSSRIIYSDDHGKTWHAGEAVNDNRQVD----------
              : .                         . :    :
Prim.cons.   3N2GRQ3SPVY2TSNVSH22GK3V3AF3YSDN2G33WTR3E3SL3232NQRI22VGQ2SI

                    550       560       570       580       590       600
                      |         |         |         |         |         |
3SILxx0      ----------LYS-----------------------GEVKLIDDFYP------------
pdb1dilA     ----------LYS-----------------------GEVKLIDDFYP------------
pdb1n1tA     GDENSGYSSVLYKDDKLYSLHEINTNDVYSLVFVRLIGELQLMKSVVRTWKEEDNHLASI
pdb2bf6A     -------GKIINSKTLSDDAPQ--------L------TECQVVEMPNG---Q--------
pdb2sliA     ------------------------------------AEAQIVEMPDG---S--------
pdb3b69A     GDENSAYSSVLYKDDKLYCLHEINSNEVYSLVFARLVGELRIIKSVLQSWKNWDSHLSSI
pdb3h73B     -------GQKIHSSTMNNRRAQ--------N------TESTVVQLNNG---D--------
                                                   *  ::.
Prim.cons.   GDENS2Y2SVLYSD2KLY4LH2IN2N2VYSLVF2RL2GE2QL23332G2WK52D2HL2SI
```

```
                    610       620       630       640       650       660
                      |         |         |         |         |         |
3SILxx0      -------KVGNASGAG------------------------YSCLSYRKN-----------
pdb1dilA     -------KVGNASGAG------------------------YSCLSYRKN-----------
pdb1n1tA     CTPVVPAXXXXXXGCGAAVPTAGLVGFLSHSANGSVWEDVYRCVDANVANAERVPNGLKF
pdb2bf6A     ------LKLFMRNLSG--------------------------YLNIATS-----------
pdb2sliA     ------LKTYLRTGSN--------------------------CIAEVTS-----------
pdb3b69A     CTPAXXXXXXXXXGCGPAVTTVGLVGFLSHSATKTEWEDAYRCVNASTANAERVPNGLKF
pdb3h73B     ------VKLFMRGLTG--------------------------DLQVATS-----------
                            .                           :
Prim.cons.   CTP222LK333R2G3G2AV2T2GLVGFLSHSA2222WED2Y2CL222TSNAERVPNGLKF

                    670       680       690       700       710       720
                      |         |         |         |         |         |
3SILxx0      --VDKETLYVVYEANGSIEFQDLSRHLPVIKSYN--------------------------
pdb1dilA     --VDKETLYVVYEANGSIEFQDLSRHLPVIKSYN--------------------------
pdb1n1tA     NGVGGGAVWPVARQGQTRRYQFANYRFTLVATVTIDELPKGTSPLLGAGLEGPGDAKLLG
pdb2bf6A     --FDGGATWDETVEKDTNVLEPY-CQLSVINYSQK---VDGKDAVIFSNPNARS------
pdb2sliA     --IDGGETWSDRVPLQGISTTSYGTQLSVINYSQP---IDGKPAIILSSPNATNG-----
pdb3b69A     AGVGGGALWPVSQQGQNQRYHFANHAFTLVASVTIHEVPKGASPLLGASLDSSGGKKLLG
pdb3h73B     --KDGGVTWEKDIKRYPQVKDVY-VQMSAIHTMH-----EGKEYIILSNAGGPK------
                .    :                 :. :
Prim.cons.   2GVDGGA2W2VY222Q2I32Q2Y2RQLSVI3S33I2E2P2GKS22I2S22N2PGG2KLLG

                    730       740       750       760       770       780
                      |         |         |         |         |         |
3SILxx0      ------------------------------------------------------------
pdb1dilA     ------------------------------------------------------------
pdb1n1tA     LSYDKNRQWRPLYGAAPASPTGSWELHKKYHVVLTMADRQGSVYVDGQPLAGSGNTVVRG
pdb2bf6A     --RSNGTVRIGLINQVGTYENGEPKYEFDWKYNKLVKP--GYYAYSCLTELSNGNIGLLY
pdb2sliA     --RKNGKIWIGLVNDTG--NTGIDKYSVEWKYSYAVDTPQMGYSYSCLAELPDGQVGLLY
pdb3b69A     LSYDKRHQWQPIYGSTPVTPTGSWEMGKRYHVVLTMANKIGSVYIDGEPLEGSGQTVVPD
pdb3h73B     --RENGMVHLARVE-----ENGE----LTWLKHNPIQK--GEFAYNSLQELGNGEYGILY

Prim.cons.   LSRDNG52WI2L224T2332TG2W2Y4K5W22VLT2A53QGS22Y22LPELG2G2TG2LY

                         790       800       810       820       830
                           |         |         |         |         |
 3SILxx0      ----------------------------------------------------------
 pdb1dilA     ----------------------------------------------------------
 pdb1n1tA     ATLPDISHFYIGGPRSKGAPTDSRVTVTNVVLYNRRLNSSEIRTLFLSQDMIGTD---
 pdb2bf6A     EGTPSEE-----------------------------MSYIEMNLKYLESG--------
 pdb2sliA     EKYDSWSRN---------E----------LHL-KDILKFEKYSISELTGQA-------
 pdb3b69A     ERTPDISHFYVGGYKRSGMPTDSRVTVNNVLLYNRQLNAEEIRTLFLSQDLIGTEAHM
 pdb3h73B     EHTEKGQN-------------------------AYTLSFRKFNWDFLSKDL-------

Prim.cons.   E5TP2ISHFY2GG2222G3PTDSRVTV2NV3LYNR4L2FEEI2TLFLSQDLIGT2AHM
```
